# Pathways to Energy‐efficient Water Production from the Atmosphere

**DOI:** 10.1002/advs.202204508

**Published:** 2022-10-26

**Authors:** Yaohui Feng, Ruzhu Wang, Tianshu Ge

**Affiliations:** ^1^ Research Center of Solar Power & Refrigeration Institute of Refrigeration and Cryogenics School of Mechanical Engineering Shanghai Jiao Tong University 800 Dongchuan Road Shanghai 200240 China

**Keywords:** atmospheric water harvesting, device fabrication, energy efficiency, heat and mass transfer, sorption, techno‐economic evaluation

## Abstract

Atmospheric water harvesting (AWH) provides a fascinating chance to facilitate a sustainable water supply, which obtains considerable attention recently. However, ignoring the energy efficiency of AWH leads to high energy consumption in current prototypes (ca. 10^1^ to 10^2^ MJ kg^−1^), misfitting with the high‐strung and complicated water‐energy nexus. In this perspective, a robust evaluation of existing AWHs is conducted and a detailed way to high‐efficiency AWH is paved. The results suggest that using cooling‐assisted adsorption will weaken the bounds of climate to sorbent selections and have the potential to improve efficiency by more than 50%. For device design, the authors deeply elucidate how to perfect heat/mass transfer to narrow the gap between lab and practices. Reducing heat loss, recovering heat and structured sorbent are the main paths to improve efficiency on the device scale, which is more significant for a large‐scale AWH. Besides efficiency, the techno‐economic evaluation reveals that developing a cost‐effective AWH is also crucial for sustainability, which can be contributed by green synthesis routes and biomass‐based sorbents. These analyses provide a uniform platform to guide the next‐generation AWH to mitigate the global water crisis.

## Graphical abstract

A uniform energy analysis platform is built to evaluate energy performance of atmospheric water harvesting (AWH). The study highlights that employing cooling‐assisted sorption and performing well‐managed heat/mass transfer are promising paths to promoting efficiency. Besides, the techno‐economic evaluation suggests that developing a cost‐effective AWH is also crucial for a sustainable future.

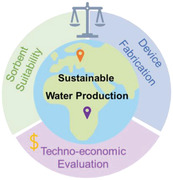

## 1 Introduction

Water is a scarce resource. Approximately 2.2 billion people live without safe drinking water globally, and the World Health Organization (WHO) predicted that half of the world's population will suffer from water shortage by 2050.^[^ ^1^, ^2^ ^]^ The pursuit of sustainable, accessible, and clean drinking water has thus pushed technical developments on freshwater production.^[^ ^3^, ^4^ ^]^ Atmospheric water harvesting (AWH), digesting ambient humidity and converting it into liquid water, features ubiquity, scalability and wide applicability, which has the great potential to fulfill the vision of the United Nation's 6th Sustainable Development Goals (SDG6).^[^ ^5^ ^]^

**Figure 1:**
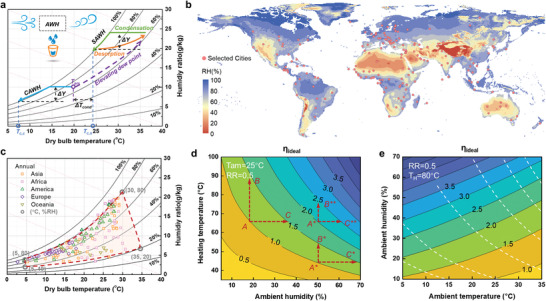
Evolutions and thermodynamic limit of atmospheric water harvesting. a) Water harvesting cycle of CAWH and SAWH on the psychrometric chart. b) Global average annual humidity map. c) Temperature and humidity in selected global main cities. Most cities are the capital and major cities of a country. The geographical locations of these cities can be found in Figure 1b and Figure S1, Supporting Information. Seasonal weather of these cities is plotted in Figure S2, Supporting Information. d,e) Ideal thermal efficiency with different recover ratios, heating source temperature, and ambient conditions. The white dash line in Figure 1e is the iso‐humidity ratio curve.

Dewing, commonly seen in nature, occurs when the surface temperature is below the dew point and is commercially developed to produce condensate water using an electricity‐driven process (e.g., refrigeration‐based system), also named cooling‐based AWH (CAWH). As shown in **Figure** **1**a, if a water production rate (*ΔY*) is specified, the feed air should be reduced to an extremely low temperature. When the ambient condition is at low humidity, it will hardly work due to the possible frost formation. Therefore, the massive electrical consumption and limited climate adaptability leave a great chance to seek an alternative energy‐efficient AWH.^[^ ^6^ ^]^ Sorption‐based AWH (SAWH), employing accessible porous structures to grab moisture from the air, is typically a thermal‐driven sustainable path.^[^ ^7^ ^]^ Through screening materials that obey the relationships between their properties (e.g., isotherms, uptake, and kinetics) and climate conditions, the dew point can be even elevated above the ambient temperature via low‐grade thermal energy (e.g., solar energy) driven‐desorption stage (see Figure 1a), making up for the shortcoming of CAWH.^[^ ^8^ ^]^

To date, the advances in material science laid a solid foundation for the implementation of SAWH, especially for the metal–organic framework (MOF) which can trap water from an extremely‐arid atmosphere.^[^ ^9^, ^10^, ^11^ ^]^ Yet, a wide gap existed from the materials level to practical application, for example, the decline of harvested water in field test was up to 50% compared with sorbent test.^[^ ^12^ ^]^ Although the popular belief deems that SAWH has the potential to relieve one billion people's drinking water stress,^[^ ^13^ ^]^ ignoring energy efficiency has resulted in stubbornly high energy consumption in most existing AWH prototypes and that will be worse when scaling up AWH. Accordingly, the limited daily water productivity is still unable to meet the people's drinking water needs (at least 4 L/day per person). With the increasing complexity of the water‐energy nexus, mastering of energy‐efficient freshwater production technology is imperative for sustainable development.^[^ ^14^ ^]^

In this perspective, we address this gap to answer—how to develop an energy‐efficient AWH toward a sustainable future? In doing so, we conduct an analysis from different efficiency viewpoints in the hope of guiding the practice of AWH. Concerning the status quo of existing AWHs and thermodynamic restrictions, we first tap the basic reasons for their high energy consumption and faced dilemmas by uncovering its efficiency limit from aspects of sorbent and device. As an action, we build a uniform energy analysis framework and identify the sorbent suitability, device fabrication and techno‐economic evaluation to break the barrier toward sustainable water production. We envision that these discussions could provide a platform for further exploration of next‐generation AWH, meanwhile, we firmly believe that with continued efforts to promote energy efficiency, utilizing atmospheric water will pump us into a water stress‐free future to facilitate the achievement of sustainable development.

## 2 Thermodynamic Restrictions of AWH

### 2.1 Theoretical Efficiency Limit on AWH

The global humidity distribution (see Figure 1b) illuminated that more than two‐thirds of lands experience a low‐humidity climate (RH < 50%). Among them, the temperature and humidity in over one hundred main cities are collected (see Figure 1c), finding that the average weather during a year is mainly concentrated in the marked red box (the temperature at 5–35 °C and humidity at 20–80%), but the extreme climate may be out of this due to the weather fluctuations (see Figure S2, Supporting Information).

Although the arid or semi‐arid regions mainly located in Asia and Africa suffer from a poor economic and infrastructure foundation (as revealed by GDP value in Figure S3, Supporting Information), abundant solar irradiations inherently exist in these water‐shortage regions,^[^ ^15^ ^]^ providing a compelling chance to quench the thirst using solar‐driven SAWH. In addition, extreme arid or cold climates in Oceania or Europe also hinder the application of CAWH (Figure S4, Supporting Information). To highlight the superiority of SAWH, the corresponding energy efficiency of existing works should be evaluated.

The thermal efficiency (Equation (1)), defined as the ratiolatent heat (*m*_w_ *h*_fg_) to required energy (*Q*_in_) for water harvesting, is a widely‐adopted energy index to evaluate the performance of solar‐driven AWH.^[^ ^16^ ^]^

(1) $$\eta =\frac{m_{w}h_{\mathrm{fg}}}{Q_{\mathrm{in}}}$$

To explore its limit, the required minimum thermal energy (*Q*_in,ideal_) needs to be evaluated, which can be determined by the thermodynamic least work (*W*_min_), as expressed by Equation (2). Assuming the heating and ambient temperature is *T*_H_ and *T*_o_, respectively:

(2) $$Q_{\mathrm{in},\mathrm{ideal}}=\frac{W_{\min}}{1-T_{o}/T_{H}}$$

The calculation of *W*_min_ can be described by the exergy change of outlet and inlet air (see Methods Section and Figure S5, Supporting Information), and the recover ratio (RR) is involved to describe how much moisture in feed air is captured (RR = 1 − Y_a,out_/Y_a,in_, where Y_a,in_ and Y_a,out_ refer to the air humidity ratio of inlet and outlet, respectively).

Accordingly, the ideal thermal efficiency *η*_ideal_ is evaluated with functions of ambient condition and heating source temperature, as mapped in Figure 1d,e and Figure S6, Supporting Information. It should be noted that the ideal thermal efficiency is limited fundamentally by the Carnot efficiency (*η*_c_ = 1 − *T*_0_/*T*_H_), thus the upper boundary is not 1. As indicated in Figure S6, Supporting Information, *η*_ideal_ is highest when RR approaches zero, which means that the process does not involve liquid water.^[^ ^17^ ^]^ More realistically for a practical AWH,^[^ ^16^ ^]^ RR of 0.5 is used to explore the influence of ambient conditions and heating temperature on the efficiency limit. The results indicate that *η*_ideal_ can reach more than 2.5 in mid‐humid climates (RH > 50%) under a common heating temperature (*T*_H_ > 70 °C), while that is lower than 1.5 in arid climates (RH < 30%), explaining that the required least work is much harsher in the arid climate than that in humid regions. Therefore, harvesting water from arid air is more challenging. From the perspective of heat‐to‐work transformation, a higher temperature could output more work, and thus the ideal efficiency can be doubled when the heating temperature changes from 60 to 100 °C. Accordingly, photothermal materials are unusually included in current AWHs.^[^ ^18^, ^19^ ^]^ In addition, Figure 1d,e also indicates that humidification is another possible way to improve efficiency. In arid climates, humidification (trajectory A–C) becomes more effective than increasing the heating temperature (trajectory A–B), but it is the opposite in humid regions (trajectory A*–B and A*–C*). The different distances between iso‐efficiency curves reveal that the influences of both these two ways can be enhanced in humid climates (trajectory A**–B** and A**–C**). Furthermore, Figure 1e shows that cooling the feed air is also beneficial to improve efficiency. Since the change of sensible temperature is accompanied by the change of RH, the improving efficiency enabled by the cooling effects (along the iso‐humidity ratio curves) is more effective in cold and humid climates than that in hot and arid climates, which can be described by the different distances between iso‐efficiency curves.

The theoretical efficiency limit accounts for the ideal water productivity under the specific solar energy input. Although such a moisture separation for AWH is similar to an air‐conditioning system for dehumidification,^[^ ^20^ ^]^ the evaluation indexes and results are quite different due to the different targets for different systems (freshwater for AWH but comfortable air for air‐conditioning). Therefore, the system design of AWH should not be carried out blindly according to the method of air‐conditioning. The key point is not to accurately control the air temperature and humidity, but the freshwater water output. Nevertheless, for SAWH and air‐conditioning, sorbent tailoring has a similar principle. The ideal superior sorbent should be high water uptake, fast sorption kinetics, low energy barrier, long‐term stability, and so on.^[^ ^21^ ^]^

### 2.2 Energy Consumption of Current AWHs

**Figure 2:**
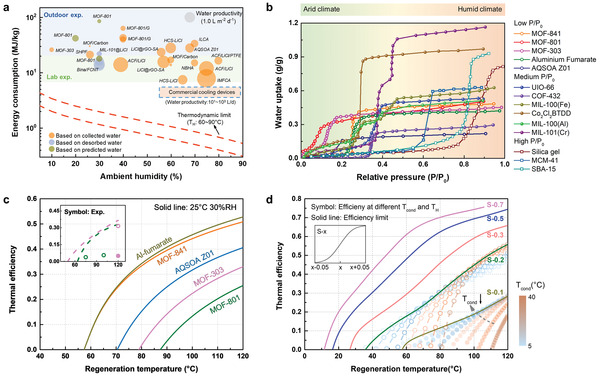
Status quo of current AWH and exploration of efficiency limit. a) Specific energy comparisons (SEC) of existing SAWH and commercial cooling‐based AWH. All the energy consumption is from solar energy. The water productivities are plotted based on the area of the circle and the unit of that is water (L) per solar energy area (m^2^) per day. The thermodynamic limit is plotted assuming the RR of 0.5 and ambient temperature of 25 °C. Detailed values can be found in Table S2, Supporting Information. b) Isotherms of common S‐shaped sorbents at 25 °C. Detailed references for specific values can be found in Figure S7, Supporting Information. c) Thermal efficiency of common sorbent used in the arid region. Solid lines are conducted in typical arid conditions at 25 °C/30%RH. The regeneration temperature refers to the sorbent temperature during the desorption stage. The inserted graph is calculated based on the literature condition (MOF‐303, adsorption at 27 °C/32%RH, desorption at 120 °C/10%RH and condensation at 10 °C; MOF‐801, adsorption at 20 °C/50%RH, desorption at 75 °C/10%RH and condensation at 20 °C). Symbols represented the experimental results (energy consumption of auxiliary equipment like condenser is not included in open cycle symbols, while included in closed symbol). The differences between the experimental results and prediction (dash line) are mainly caused by heat loss and poor mass transfer. d) Thermal efficiency limit of sorbents with different isotherms. The S‐x (x = 0.1 to 0.7) represents that the center position of step locates at the relative pressure (P/P_0_) of x (see Figure S8, Supporting Information). It should be noted that the purpose of using fitting isotherms is to investigate the impacts of step pressure, not to simulate the real‐sorbent isotherms. Symbols for S‐0.1 and S‐0.2 refer to thermal efficiency at different temperatures and limit lines are drawn according to the boundary of these symbols (see Figures S10 and S11, Supporting Information).

Compared with the thermodynamic limit, we then summarized the specific energy consumption (SEC = *Q*_in_/*m*_w_, it also can be derived from thermal efficiency easily) of existing AWHs.^[^ ^9^, ^12^, ^18^, ^22^, ^23^, ^24^, ^25^, ^26^, ^27^, ^28^, ^29^, ^30^, ^31^, ^32^, ^33^, ^34^ ^]^ For passive AWH, the energy input is from solar energy but the active AWH needs extra electricity demands. For a fair comparison, we unified all energy as solar energy, as shown in **Figure** **2**a. The conversion efficiency from solar to electricity is assumed to be 20% using a photovoltaic (PV) panel, and the electrical heater used in some works is considered that it can be replaced by the solar collector with an efficiency of 70%.^[^ ^13^, ^27^ ^]^ As seen, the SEC of most lab experiments (≈10^1^ MJ kg^−1^) are lower than the outdoor tests (up to 10^2^ MJ kg^−1^) owing to the relative standard and stable environment in the lab and uncertain irradiation and weather outdoor, yet all results are far away from the thermodynamic limit (beyond 1∼2 orders of magnitude). Generally, the energy consumption based on collected water is higher than that based on predicted or desorbed water. As indicated in Table S1, Supporting Information, low release and condensation efficiency reveal that not all adsorbed water can be released or collected. Therefore, the condensation process is of importance for device performance. Early works used gram‐scale sorbent to prove the feasibility of AWH, thus little water was condensed and collected. For a practical application, the performance evaluation should consider the collected water instead of the predicted value, because such a value is more intuitive and useful.

Another important index is water productivity (L m^−2^ d^−1^, liter water per solar area per day) for solar‐driven AWH. Even though current works have promoted this index greatly by optimal device management or super‐moisture sorbent,^[^ ^25^, ^31^, ^35^ ^]^ it is still lower than 3.5 L/(m^2^d). Comparably, CAWH, which has been already commercialized, is closer to the thermodynamic limit with slightly higher water productivity (up to 10^3^ L/d, equivalent to 3–6 L/(m^2^d) but losing the compactness) than SAWH.^[^ ^36^, ^37^ ^]^ It is worth noticing that such values are not the limit of CAWH due to the ongoing development of PV technology, and scaling up the SAWH cannot keep the constant productivity and energy consumption. With the increasing utilization of renewable energy, the active AWH can use more sustainable technologies to meet energy demands. To this end, pushing the high‐efficient SAWH technology to the commercialization road, especially in arid climates, is still warranted and has a long way to go.

To find the reason behind low efficiency in current works, we identify the thermal efficiency using different sorbents. Energy consumption could be expressed as the sum of latent (*Q*_lat_) and sensible heat (*Q*_sen_) required for a specific sorbent:

(3) $$Q_{\mathrm{in}}=Q_{\mathrm{lat}}+Q_{\mathrm{sen}}$$

Latent heat is attributed to the adsorption enthalpy, and the sensible heat comes from the energy for heating sorbents, vapor and air (see Supporting Information). Thus, the actual thermal efficiency must rely on the performance of sorbents. Then isotherms of common sorbents^[^ ^9^, ^12^, ^22^, ^38^, ^39^, ^40^, ^41^, ^42^, ^43^, ^44^, ^45^ ^]^ are summarized in Figure 2b. Among them, the sorbents with earlier steps (step at low pressure) obtain an enormous interest in arid regions and have been demonstrated in a real‐world scenario, like MOF‐303,^[^ ^11^, ^12^ ^]^ MOF‐801,^[^ ^9^, ^24^ ^]^ AQSOA Z01^[^ ^22^ ^]^ and Al‐fumarate,^[^ ^12^ ^]^ to name a few. Thermal efficiencies of these sorbents are shown in Figure 2c at a typical arid condition (25 °C and 30%RH). As indicated, even though the water uptake of AQSOA Z01 is lower than MOF‐303 and MOF‐801, the thermal efficiency of AQSOA Z01 is higher than others, providing evidence that efficiency is tightly impacted by the stepwise position due to the potential of low‐temperature regeneration (Figure S9, Supporting Information). To eliminate the effects of water uptake (and other thermal properties like adsorption enthalpy), all properties of sorbents are unified except the stepwise position, and related thermal efficiency limits under global experienced weather (Figure 1c) are investigated as displayed in Figure 2d. The results also elucidate that the thermal efficiency of sorbent with later step (step at high pressure) is much higher than that of sorbent with early step. However, the fact is that we are obliged to choose sorbent with an earlier step to adapt to arid climate in practice.

Based on these analyses, one of the dilemmas on AWHs can be drawn, that is: i) efficiency lifting using sorbent with a later step is incompatible with its suitability in arid climates; and beyond that, the differences between the predicted thermal efficiency and actual experimental results (insert graph in Figure 2c) indicate that ii) current AWHs suffer from poor heat management or mass transfer on device scale,^[^ ^16^, ^18^ ^]^ which could be another optimization direction. Therefore, to promote the high‐efficiency AWH, two questions should be answered based on the thermodynamic restrictions: i) how to use the sorbent with later step in the arid climate? and ii) how to perform better heat and mass transfer on device scale? Herein, we try to rationalize these mismatched relationships from the aspects of sorbents suitability and device fabrication, as discussed in the following.

## 3 Pathway to High‐Efficiency AWH

### 3.1 Broaden the Sorbents Suitability

**Figure 3:**
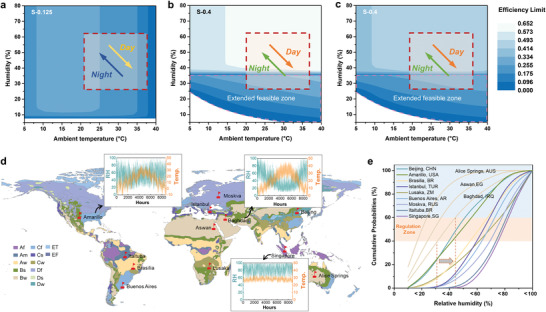
Efficiency limit of sorbents with different isotherms and superiority of CSAWH. a) Efficiency limit of sorbent S‐0.125 which represents the common MOF‐303 in a desert climate (T_H_ = 100 °C). b) Efficiency limit of sorbent S‐0.4 which represents the sorbent with moderate step (T_H_ = 100 °C). The feasible zone is extended using CSAWH. The efficiency in the extended zone (marked in magenta color) is calculated based on the optimal adsorption temperature (Figure S13, Supporting Information). Efficiency maps for other types of sorbents can be found in Figure S14, Supporting Information. c) Efficiency limit of sorbent S‐0.4 at a heating temperature of 70 °C. d) World map of Koppen–Geiger climate classification and annual transient weather data of several representative cities. Symbols refer to tropical rainforest (Af), tropical monsoon (Am), tropical savannah (Aw), arid desert (Bw), arid steppe (Bs), temperate dry summer (Cs), temperate dry winter (Cw), temperate without dry season (Cf), cold dry summer (Ds), cold dry winter (Dw), cold without dry season (Df), polar tundra (ET), and polar frost (EF). Other transient weather data besides Baghdad, Amarillo and Singapore can be found in Figure S15, Supporting Information. e) Cumulative probabilities of humidity range based on the transient weather data. The humidity distribution histogram can be found in Figure S16, Supporting Information.

Derived from Figure 2d, the efficiency limit of sorbent S‐0.125 (representing the common MOF‐303 mostly used in arid regions) is mapped in **Figure** **3**a (*T*_H_ = 100 °C). Owing to the weather fluctuation featured with low temperature/high humidity at night and high temperature/low humidity at day, the S‐0.125 could be a good choice, but its productivity and thermal efficiency are limited. Recent practices proved that a multicyclic AWH with adsorption in the daytime can promote productivity greatly,^[^ ^12^, ^25^, ^31^ ^]^ while the efficiency map (Figure 3a) indicates that it will be worse with much lower efficiency (0.176 at 35 °C/30%RH) during the daytime. Traditional routes of improving efficiency from the aspect of the sorbents are to tailor a sorbent with high uptake and reduced adsorption enthalpy,^[^ ^46^ ^]^ while their impacts are limited and they are painstaking (Figure S12, Supporting Information). In this context, how to flexibly broaden the suitability of sorbent with later steps is of vital importance.

Inspired by the opposite air humidity trends with temperature fluctuation, cooling‐assisted sorption (CSAWH) may be a possible way to help the SAWH out of straits.^[^ ^43^ ^]^ Once a cooling source is employed in the adsorption phase, the effective RH near the sorbent is elevated and thus the equilibrium on the low humidity is broken. Consequently, the sorbent can work in the low‐humidity region. For instance, from a conventional view, S‐0.4 (representing the sorbent with moderate step like MIL‐101, *T*_H_ = 100 °C) is not suitable due to the infeasibility in low‐humidity regions for SAWH, as displayed in Figure 3b (outside the marked zone), while its feasible zone is expanded via employing CSAWH (considering a cooling source come from a vapor compression cycle with COP of 5,^[^ ^47^, ^48^ ^]^ and the electricity demands are converted into solar energy). Nevertheless, the thermal efficiency of S‐0.4 in arid climates is still competitive with that of S‐0.125. Moreover, when toward a multicyclic AWH at daytime with low humidity (35 °C/30%RH), the efficiency (0.31) is still higher than S‐0.125 (0.176), despite extra cooling demand and low solar‐to‐electricity efficiency. With the fluctuation of weather, the efficiency of S‐0.4 is as higher as 0.61 than that of S‐0.125 (0.25) at 25 °C/60%RH.

Another convincing evidence is that low‐temperature regeneration comes true in arid regions using CSAWH. Figure 3c further proves that even if the heating temperature is as low as 70 °C using S‐0.4, its efficiency is still higher than that of S‐0.125 at 100 °C. In addition, the cooling capacity in adsorption can be recovered to reduce the condensation temperature, then the desorption temperature can be further declined. Thus the heat pump should be another choice to meet the demands of the heat source.^[^ ^49^ ^]^

Grasping energy from nature, some recent progress utilized radiative cooling (RC) to provide a cooling source to fabricate a passive CSAWH.^[^ ^32^, ^50^ ^]^ To this end, the extra cooling energy for adsorption is free from the sky and the cooling capacity could even achieve 145 W m^−2^ because the arid climate (low humidity) is beneficial to improving cooling capacity. Although the effect of radiative cooling can give a sub‐ambient temperature with a limit temperature difference of ≈8 °C,^[^ ^51^ ^]^ there is nearly no cooling capacity at this temperature difference. In this case, the RC method is more suitable to improve water uptake of sorbents with linear isotherms or the condition that ambient humidity is near to the step for S‐shaped isotherms.^[^ ^32^, ^43^ ^]^

With the climate fluctuation diurnally and seasonally, the limitation of extreme weather to the sorbent will be weakened using CSAWH, but sorbents scanning still should be highly contextualized. Figure 3d shows the world map of Koppen–Geiger climate classification and several representative transient weather data. Cities with arid desert climates such as Baghdad, Aswan and Alice Springs featured fluctuating weather, but the probability of extreme‐low humidity (20–40%RH) remains few, as per the statistical data in Figure 3e. When employing CSAWH using S‐0.4 (e.g., MIL‐101, UiO‐66, and COF‐432), the suitable probability will be extended from 40% to 60% for Baghdad. By contrast, tropical cities like Singapore experience relatively stable weather with high humidity, then the effect of CSAWH is not significant, thus the hydrogel could be a better choice.^[^ ^52^ ^]^ For cities with probability curves between the above two, like arid stepper region (Amarillo), cold dry winter (Beijing) and so on, sorbent with later step such as MCM‐41 is more suitable. With the extended feasibility using CSAWH, prolonging the high‐efficiency work time provides a great chance for multicycle AWH in the daytime.

### 3.2 Well‐Managed Heat/Mass Transfer on Device Fabrication

The gap between the predicted and experimental thermal efficiency indicates that optimizing heat and mass transfer in device fabrications is another way to improve efficiency (Figure 2c), but the device design is often neglected. In this field, reducing heat loss (*Q*_loss_) is the most effective approach, and thus the thermal efficiency also can be expressed as:

(4) $$\eta =1-\frac{Q_{\mathrm{loss}}}{Q_{\mathrm{in}}}$$

The unreasonable design will lead to a large heat loss from the device to the ambient. The heat loss includes convection, conduction and radiation. Simply, the total heat loss can be uninformed as *Q*_loss_ = K*AΔT*, where the parameters K, *A* and *ΔT* refer to the overall heat transfer coefficient, area of heat transfer and temperature difference. In this case, decreasing *ΔT* or increasing thermal resistance using a well‐insulated wall, selective solar absorber and glass cover is a common practice. However, there is no universal design guideline for optimizing thermal management so far.

**Figure 4:**
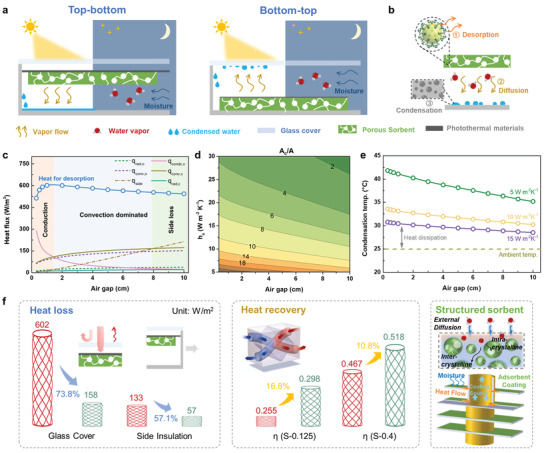
Heat and mass transfer in AWH device. a) Classifications of passive SAWH, consisting of top‐bottom and bottom‐top types based on the vapor flow direction. b) Mass transportation process in the desorption phase. c) Heat flux varied with the distances of the air gap. The size of sorbent, solar‐adsorber and glass cover is assumed at the same size (30 × 30 cm), and the incident solar flux is evaluated at one sun (1000 W m^−2^). The ambient temperature is 25 °C and the condensation temperature is fixed at 30 °C. d) Ratio of required heat dissipating area to solar irradiation area. e) Evolution of condensation temperature at different heat convection coefficients. f) Roadmap for optimal heat and mass transfer. The red and blue columns represent the without and with optimization (e.g., glass cover, well insulation, and heat recovery). The thermal efficiency is evaluated at the heating temperature of 100 °C for S‐0.125 (adsorption at 25 °C/30%RH) and 70 °C for S‐0.4 (adsorption at 25 °C/45%RH). The coefficient of heat recovery is defined as the ratio of the air temperature difference between the sorbent inlet and outlet to the difference between the sorbent outlet and condenser outlet.

Passive SAWH can be categorized into top‐bottom and bottom‐top based on water vapor flow direction, as shown in **Figure** **4**a. The related energy flows are depicted in Figure S17, Supporting Information, similarly to passive desalination.^[^ ^53^ ^]^ In general, incident solar flux passes through the glass cover to heat the sorbents. A selective absorber can be used to reduce the emission in the infrared region and enhance the absorption in the solar spectrum, and installing thermal insulation is essential to avoid significant heat loss from the side wall.^[^ ^22^, ^35^ ^]^ During the desorption phase, the water vapor escapes from the pore of sorbents under the driven force of humidity difference. The desorbed water vapor further diffuses to the condensation surface, followed by the nucleation and growth of droplets (Figure 4b).

However, the different designs need different considerations. For bottom‐top designs, photothermal materials need to be involved to develop the hybrid or composite sorbents in order to guarantee the photothermal effect. In addition, the vapor diffusion in the desorption‐to‐condensation process has the assistance of buoyancy. However, the condensate droplet should be removed timely to avoid reduction of net incident solar flux by water film. Meantime, the high temperature at the top cover may cause the water re‐evaporation, and lead to a high humidity environment near the sorbent, preventing the desorption process. Thus, most works install the glass cover in a slope to remove the droplet via gravity.^[^ ^34^, ^54^ ^]^ For the top‐bottom type, the sorbents unusually need to be coated or linked on the backside of the solar absorber to get the solar energy during the desorption phase, but the natural buoyancy effect may increase the diffusion resistance.

A quantitative analysis of energy balance (see Figure 4c, top‐bottom AWH as an example, details can be found in Supporting Information) indicates that optimizing the air gap should be a key point. At a narrow gap (<1.4 cm), heat transfer between the sorbent with high temperature and the condensation surface with low temperature is mainly through conduction. With the gap increasing, heat convection becomes dominant (1.4–8.0 cm) and then the heat loss from the side‐wall is rises (>8.0 cm). As a result, the heat for desorption (describing how much heat is utilized to desorb water) appears a trend of increasing first and then decreasing.

For a condensation process, the dissipated heat to the ambient should be dispelled promptly. Besides using the soil as a huge heat sink,^[^ ^18^ ^]^ fins also can be utilized to enhance heat dissipation.^[^ ^43^ ^]^ To this end, the ratio of the required condensation area to the sorbent area (*A*_C_/*A*) can be determined, as shown in Figure 4d. Compared with natural convection (ca. 10 W m^−2^ K^−1^), forced convection with a high convective heat transfer coefficient reduces the area needed for heat dissipation, and the corresponding condensation temperature is closer to the ambient temperature (Figure 4e).

A roadmap for optimizing heat and mass transfer is illuminated in Figure 4f. For heat loss management, posing a glass cover could cut down the heat loss of 73.8% from the sorbent to ambient and a well‐insulated wall (thickness of 10 mm and heat conductivity of 0.05 W m^−1^K^−1^) could reduce heat loss by 57.1% compared with poor insulation (thickness of 5 mm and heat conductivity of 0.19 Wm^−1^K^−1^). For good measure, heat recovery should be another focus, especially for the active AWH. Due to the large temperature difference between sorbent and condenser, the heat loss is conspicuous. Assuming a coefficient of heat recovery of 0.8, the thermal efficiency of S‐0.125 and S‐0.4 could improve by 16.8% and 10.8%, respectively. Coupled heat and mass transfer is also a distinguishing feature, which has been revealed in the literature^[^ ^55^, ^56^ ^]^ and should be given more consideration, especially from lab to large scale. From this view, using structured sorbents could reduce the mass transfer resistance and enhance heat transfer, like hierarchical pore structure, honeycomb structure and sorbent coating.^[^ ^29^, ^57^ ^]^ Besides the sorbent scale, recent work has exemplified minimizing the water diffusion channel to reach rapid water sorption on the pore scale,^[^ ^34^ ^]^ which provides a basic optimization for better mass transfer.

## 4 Techno‐Economic Perspective

Improving the efficiency of AWH has been carried out by continuous efforts recently, however, facing a sustainable future, another question is raised—what should we focus on besides efficiency? For a large‐scale implementation and pursuit of sustainable water production, developing a cost‐effective AWH is equally crucial beyond efficiency, even if water is not a typical commodity.^[^ ^14^ ^]^

**Figure 5:**
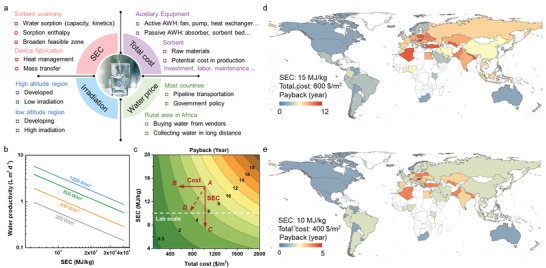
Techno‐economic evaluation of SAWH. a) Impact factors of techno‐economic evaluation. b) Water productivity varies with energy consumption using different solar fluxes. c) Payback as a function of energy consumption per liter of water and total cost per area, compared with bottled water price ($0.3/L). Average solar irradiation is assumed as 600 W m^−2^ within 8 h per day. d,e) Payback maps of AWH by countries compared with the local price for drinking water. The solar irradiations by countries are calculated based on the long‐term average of global horizontal irradiations (GHI). In many African areas, drinking water is mainly from collecting or buying commercial water from long‐distance water resource or vendors, resulting in the blank area in these countries. Such drinking water is more expensive and the quality cannot be ensured.

The main concerns for techno‐economic are marked in **Figure** **5**a. In addition to energy consumption, the actual solar irradiation, total cost and local water price are major factors. Herein, we give a uniform techno‐economic evaluation including these factors, as expressed in Equation (5):

(5) $$\mathrm{Packback}\left(d\right)=\frac{C_{\mathrm{total}}\mathrm{SEC}}{I_{\mathrm{irr}}P_{\mathrm{water}}}$$

where *C*_total_, *I*_irr_ and *P*_water_ represent the total costs in AWH fabricating process ($/m^2^), solar irradiation (Wh m^−2^ d^−1^) and water price ($/L).

The total cost should include the materials, investment, labor, manufacturing and maintenance costs, and so on. Particularly, the materials cost should consider the potential cost from the production process (solvent consuming, washing, centrifugation, filtration, drying, and so on) rather than just raw materials. For example, the process cost occupied ca. 40–90% of total production costs for MOF,^[^ ^58^ ^]^ thus existing works underestimated the sorbent cost by only calculating the salt and linker, while such costs can be reduced by a scaling‐up production.^[^ ^59^ ^]^ For the passive solar‐driven AWH, the costs are mainly from the sorbents. Therefore, sustainable sorbents need reasonable selection considering their toxicity, renewability, and eco‐friendliness since these factors are vital for environmental sustainability and human health.^[^ ^31^ ^]^ Nowadays, developing a sustainable, facile and scalable synthesis route of MOFs has been widely investigated,^[^ ^60^ ^]^ and biomass‐based sorbents showed clear environmental and economic advantages for a sustainable future.^[^ ^31^ ^]^ For an active AWH, the total costs are also contributed from the auxiliary equipments like air fans, solar collectors, heat exchangers, pumps, and so on, but the high‐water productivity could balance these costs.

As indicated in Figure 5b, higher water productivity could be output at higher solar irradiation. Most developed countries located in the high‐altitude regions with low solar irradiation, and low‐altitude developing countries received higher irradiation but suffer from severe water crises. Assuming a practical average solar irradiation of 600 W m^−2^ for 8 h and the total cost is below $2000/m^2^ (which is promising for commercial fabrication^[^ ^61^ ^]^), the payback of AWH is drawn in Figure 5c, compared with the moderate price of bottled water ($0.3/L), revealing that reducing cost (trajectory A–B) is more sensitive than reducing SEC (trajectory A–C), while the effect of reducing these two factors at the same time is more significant (trajectory A–D).

However, in a real‐world scenario, solar irradiation relies on solar altitude angle and actual weather, and water price is decided by government policy and actual drinking way. A payback map is determined based on the global horizontal irradiation (GHI) and drinking water price by counties,^[^ ^15^, ^62^ ^]^ as shown in Figure 5d. The shorter paybacks (around one year) in USA, Norway and Canada are contributed by local higher water prices, even though irradiation is relatively low. Although abundant irradiation existed in low‐altitude countries, the current lower water price led to a longer payback. Thus, developing an energy‐efficient and cost‐effective AWH has a clear benefit, as mapped in Figure 5e. Assuming that the larger scale of AWH could be designed to be consistent with the current lab level (10 MJ kg^−1^) with a better heat/mass transfer design and lower cost, the global maximum payback will be shortened to 5 years.

However, in Sub‐Saharan Africa (blank areas in Figure 5d,e), there are no perfect infrastructures for drinking water, buying water from vendors or going out to find water resources are the main manners to obtain drinking water, resulting in lost work or study chances for women and children besides insurable water quality, and then they fall into an endless loop of poverty.^[^ ^63^ ^]^ Therefore, the water price there is not the decisive factor for whether to use AWH, and developing AWH is more meaningful, aiming at creating a clean water source, not just providing water. Installing an off‐grid, decentralized and portable AWH will be one of the basic infrastructures in poverty areas, along with avoiding long‐distance pipeline transportation, empowering families, increasing woman's influence and decreasing premature deaths.^[^ ^64^ ^]^

## 5 Conclusion and Outlook

In this perspective, we analyze the thermodynamic restrictions on AWH and the current level of energy consumption in reported works. Based on the results, the detailed pathways for improving efficiency are proposed from the aspects of sorbent suitability, device fabrications, and techno‐economic evaluation. Our analysis leads to three important implications for the implementation of energy‐efficient, cost‐effective and sustainable freshwater production from the atmosphere, and would provide a uniform platform to guide the practical applications in future scientific exploration and commercial development.

First, besides developing sorbents with high capacity and low energy barrier, CSAWH—employing a cooling source in the adsorption phase—could broaden the suitability of specific sorbents, providing a chance to use the sorbent with the potential of low‐temperature regeneration in fluctuating climate. In addition, this flexible regulation strategy also leaves great room to use the high‐efficiency heat pump and sky radiative cooling to ease the energy demands.

Second, reducing heat loss, performing a heat recovery and shaping materials are the main ways to maximize energy efficiency from the view of heat and mass transfer. Existing water harvesters always ignored the thermal design of prototypes, resulting in large differences from powder tests and lab devices to scale‐up AWH. Deeply understanding coupled and complicated heat and mass transportation is a more realistic concern from lab to practice.

Last but not least, developing a cost‐effective AWH is more attractive for most practical scenarios. But the necessity of installing AWH cannot be measured only by local water price, especially in Sub‐Saharan Africa. The costs estimated should include all potential costs in the production process which account for a large proportion of total costs and cannot be neglected. Pushing a green synthesis route and utilizing biomass‐based sorbents have clear sustainable advantages.

Although encouraging progress has been made recently, further explorations still should be tapped. For instance, the optimal water diffusion channel from the different scales can make a helpful contribution to enabling the rapid sorption/desorption rate. On the pore scale, both the pore distributions and pore diameters are the important factors on diffusion resistances, and that can be evaluated by the density functional theory (DFT) method.^[^ ^34^ ^]^ In this scale, a deep understanding of molecular‐level interactions could provide a vivid description of water sorption mechanisms. Recently, the single‐crystal X‐ray diffraction method was performed to reveal the evolution of water sorption behavior on MOF, creating a great opportunity to optimize the water sorption path.^[^ ^11^ ^]^ Besides, the reported photomolecular effect and sorption kinetics at the liquid–vapor interface in hydrogel may bring new inspirations about evaporation‐based applications.^[^ ^65^, ^66^ ^]^ These theoretical or experimental investigations for the vapor–liquid (such as water clusters) or liquid–solid interactions can not only guide the sorbent tailoring, device fabrications and mode innovations, but also stimulate new insights into water production, dehumidification and water treatment. When it comes to the macro view, the external forced air flow also can speed the water sorption process due to the reducing external diffusion resistance on the sorbent scale, but the extra energy consumption caused by the auxiliary components will be introduced. It should be considered that using renewable energy (such as solar or wind energy) could provide the power toward decentralized water production in remote areas. Besides, the device‐scale design should be given more attention, especially for multicyclic and continuous AWH. Achieving a 24‐hour and all‐weather AWH will greatly increase the daily water production, in this case, radiative cooling will provide a help.^[^ ^67^ ^]^ Furthermore, the condensation process should receive more attention. Employing a hydrophobic coating can remove droplets more effectively but nucleation is becoming difficult. A hydrophilic surface is conducive to providing sufficient nucleation sites but the film condensation should be avoided. In this case, developing a slippery surface or hybrid hydrophobic/hydrophilic coating is another better choice.^[^ ^68^ ^]^ However, focusing too much on a single side (sorbent or device) even cannot improve efficiency or productivity owing to the highly coupled effects on both sides. That is the reason why the performance of AWH with optimal sorbent is not comparable with optimal device design, and vice versa. Therefore, using super‐adsorbing sorbent in a well‐designed prototype can obtain more significant performance, which is always our pursuit and needs close cooperation between materials science and thermal engineering.

We believe that as a potential route toward a future with sustainable water production, AWH provides a compelling chance to utilize the vast abundance nature resource to create the source of life. In the contemporary context of water shortage and intensive energy problems, seeking energy‐efficient and cost‐effective AWH should become a pursuit to alleviate the negative impacts of water shortage on the economy, society, ecology, health and human well‐being.

## 6 Methods Section

### 6.1 Global Weather Data

The global weather data is carried out from the commercial soft Meteonorm 8.0. Main cities were selected from the main countries in Asia, Africa, America, Europe, and Oceania (marked in Figure 1b). The average dry bulb temperature (*T*_a_) and dew point (*T*_d_) were calculated based on the contemporary record during 2000–2019. Then the relative humidity (RH) and humidity ratio (*Y*_a_) are calculated according to the following equations:

(6) $$\begin{array}{c} \mathrm{RH}=\exp\left(\frac{17.27\cdot T_{d}}{237.7+T_{d}}-\frac{17.27\cdot T_{a}}{237.7+T_{a}}\right)\times 100 \end{array}$$

(7) $$\begin{array}{c} p_{\mathrm{vs}}=\exp\left(\frac{c_{0}}{T_{a}}+c_{1}+c_{2}T_{a}+c_{3}T_{a}^{2}+c_{4}T_{a}^{3}+c_{5}\ln T_{a}\right) \end{array}$$

(8) $$\begin{array}{c} Y_{a}=622\frac{\mathrm{RH}\cdot p_{\mathrm{vs}}}{101325-\mathrm{RH}\cdot p_{\mathrm{vs}}} \end{array}$$

where the *p*_vs_ is the saturated vapor pressure at temperature of *T_a_*, and the constants are *c*_0_ = −5800.2206, *c*_1_ = 1.3914993, *c*_2_ = −0.0048640239, *c*_3_ = 4.1764768 × 10^−5^, *c*_4_ = −1.4452093 × 10^−8^ and *c*_5_ = 6.5459673.

The annual average temperature and humidity is plotted in Figure 1c. Monthly average values (January, April, July, and October to present four seasons) are plotted in Figure S2, Supporting Information.

The transient weather data was conducted with the minimum resolution of 1 h. Then the data of several representative cities with different climate characteristic (marked in Figure 3d) during the 8760 h in a year was determined as in Figure S15, Supporting Information. The cumulative probabilities curves (Figure 3e) were determined based on the statistical data in hours per 10%RH range in Figure S16, Supporting Information.

### 6.2 Thermodynamic Limit

The thermodynamic limit of AWH was calculated based on the exergy analysis. The air treatment process was regarded as a box, with inlet air, outlet air and liquid water (Figure S5, Supporting Information). The required least work could be derived assuming an exergy loss of zero.

(9) $$W_{\min}=m_{a}\Delta e_{a}+m_{w}\Delta e_{w}$$

where *m*_a_ and *m*_e_ represent the mass of air and water, which obey the mass conservation. Then, the recovery ratio (RR) is defined to describe the relationship between harvested water and feed air.^[^ ^17^ ^]^ Considering solar energy to drive AWH, the ideal heat‐to‐work conversion by a Carnot cycle was assumed to calculate the ideal thermal efficiency (Equation (2)). The detailed calculation and related parameters can be found in the Supporting Information.

### 6.3 Specific Energy Consumption

The SEC in the literature is plotted in Figure 2a. Some work did not show this index; thus, the energy consumption was calculated according to the reported data (based on the desorption time, solar flux, solar utilization area, and collected water). However, some works did not collect liquid water, the reported desorbed water or predicted water was used as remarked. The ambient humidity was located based on the average humidity during the adsorption. To give a fair comparison, the solar‐to‐electricity efficiency was assumed as 20% using a PV panel, while the electric heater in the literature was considered that it could be replaced by the solar collector with an efficiency of 70%. The experimental results for sorbents using direct sunlight were assumed to have 100% conversion efficiency from sunlight to heat.

### 6.4 Thermal Efficiency

For a CAWH, the cooling capacity was calculated based on the enthalpy difference between the inlet and outlet air. The outlet air temperature was assumed as same as condensation temperature and the humidity was 100%RH. For a traditional cooling process, the condensation temperature was fixed at 4 and 10 °C, which is consistent with the HVAC process. While the optimal condensation temperature could be determined according to a water harvesting rate (5 g kg^−1^ DA and 8 g kg^−1^ DA, DA means dry air), thus the energy efficiency could be improved. Then the cooling capacity was first converted into required work using a vapor compression cycle with COP of 5.0 and then uniformed as the required solar energy using a PV (solar‐to‐electricity efficiency was assumed as 20%). The detailed equations and related parameters can be found in the Supporting Information.

For a conventional SAWH, only the energy was consumed at the desorption stage. Then the energy consumption could be divided into sensible and latent heat with different heating temperature. To eliminate the impact of maximum water uptake, it was unified as 1.0, and the effects of that were then investigated. The thermal efficiency was derived at the different ambient conditions and then the limit efficiency was mapped according to the efficiency under different conditions. When a cooling source was introduced in the adsorption phase, the cooling capacity was calculated assuming the RR of 0.5, consisting of latent and sensible heat during the cooling and adsorption process. Then the cooling capacity was converted into primary solar energy on the basis of a vapor compression cycle with COP of 5 and PV with the efficiency of 20%. The limit efficiency during the adsorption, the adsorption temperature was optimized under the boundary of maximum efficiency. The detailed equations and related parameters can be found in the Supporting Information.

### 6.5 Energy Balance on Device

Energy balance was calculated using an example of conventional passive AWH configurations. The areas of glass cover, solar absorber, sorbent, and the internal surface of the condenser were the same (square: 30×30 cm). The transmissivity and emissivity of the glass cover were 0.95 and 0.05, respectively. The external and internal convective heat transfer coefficient was assumed as 10 and 2 W m^−2^ K^−1^. The condenser was fabricated using aluminum with an emissivity of 0.05. The detailed equations and related parameters can be found in the Supporting Information.

## Conflict of Interest

The authors declare no conflict of interest.

## Authors Contribution

Y.F. conceptualized this study, conducted the analysis, and wrote the original manuscript. R.W. and T.G. took the lead in writing. All authors contributed to the manuscript structure and proof reading.

## Supporting information

Supporting Information
