# Supplementary material for: Pathways to Energy‐efficient Water Production from the Atmosphere
Source: Adv Sci (Weinh). 2022 Oct 26;9(36):2204508. doi: 10.1002/advs.202204508 (PMC9798993; doi:10.1002/advs.202204508)
Supplement: Supplementary file 1 — Supporting Information [file ADVS-9-2204508-s001.pdf]

Supporting Information

Pathways to Energy-efficient Water Production from the Atmosphere

*Yaohui Feng, Ruzhu Wang and Tianshu Ge**

Supporting Methods

S1. Thermodynamic limit

The thermodynamic limit could be calculated based on a thermodynamic analysis. The basic principle of AWH is analogous to HVAC (heating, ventilation and air-conditioning) system. Both of them can be regarded as an air treatment box, feeding with air and producing air and water[1], as shown in Supporting Figure S5. The thermodynamic least work can be expressed as the exergy difference between inlet and outlet, as expressed as

$$W_{min} = m_a \Delta e_a + m_w \Delta e_w \tag{S1}$$

where the m and e mean the mass and specific exergy, and subscripts a and w present the air and water, respectively.

Mass of air and water should be satisfied with mass conservation as

$$m_a\left(Y_{a,in} - Y_{a,o}\right) = m_w \tag{S2}$$

where the $Y_{a,in}$ and $Y_{a,o}$ refer to the humidity ratio of inlet air and outlet air.

For a thermal-driven AWH, the required minimum thermal energy can be obtained assuming a Carnot cycle existing at heat source (T_H) and heat sink (T_0), as expressed,

$$Q_{in,ideal} = \frac{W_{min}}{1 - T_o/T_H} \tag{S3}$$

Thus, the ideal thermal efficiency can be derived as

$$\eta_{ideal} = \frac{h_{fg}}{\left[\Delta e_a/\left(Y_{a,in} - Y_{a,o}\right) + \Delta e_w\right]/(1 - T_o/T_H)} \tag{S4}$$

The specific flow exergy of moist air and liquid water can be determined as

$$e_a = \left(c_{p,a} + Y c_{p,v}\right)T_0\left(\frac{T}{T_0} - 1 - ln\frac{T}{T_0}\right) + (1 + 1.608Y)R_a T_0 ln\frac{p}{p_0} + R_a T_0\left(ln\frac{1 + 1.608Y_0}{1 + 1.608Y} + 1.608Y ln\frac{Y(1 + 1.608Y_0)}{Y_0(1 + 1.608Y)}\right) \tag{S5}$$

$$e_w = h_f(T) - h_g(T_0) - T_0\left[s_f(T) - s_g(T_0)\right] + [p - p_{sat}(T)]v_f(T) - R_v T_0 ln\varphi_0 \tag{S6}$$

The first, second and third terms of right-hand side (RHS) in Equation S5 and S6 represent thermal, mechanical and chemical exergy of air and water, respectively, which explained the temperature, pressure and chemical compositions difference between the calculated state and dead state. Here, the ambient condition is selected as the dead state.

The difference between HVAC and AWH is that the target is different-comfortable air for HVAC but clean water for AWH. Thus, the normalized least work is quite different (W_{min}/m_a for HVAC but W_{min}/m_w for AWH). Consequently, the results are opposite[2]. Specifically, a

high humidity environment is beneficial to water harvesting but harsh to dehumidification to a comfortable zone.

S2. Energy consumption

Energy consumption is summarized in Figure 2a, which is conducted from the literature work. For common passive SAWH, the energy consumption for water harvesting can be calculated as

$$E_{de} = \frac{q_{sun,ave}At}{m_w} \tag{S7}$$

where $q_{sun,ave}$, A and t are average incident solar flux (W/m^2), solar irradiation area (m^2) and desorption time (s).

Besides, some works did not report thermal efficiency instead of the above parameters. Then the energy consumption for water harvesting can be estimated as

$$E = \frac{m_w h_{fg}}{\eta} \tag{S8}$$

However, some works consume electricity for active AWH and auxiliary equipment like air fans and heaters, thus it is difficult to directly compare them. Herein, the solar-to-electricity efficiency is assumed as 20% for auxiliary equipment like the condenser and air fans and commercial cooling-based AWH. Some works used the electrical heater to regenerate sorbent, thus the energy consumption is converted into solar energy using a solar collector with the efficiency of 70%.

The detailed results are shown in Supporting Table 3.

S3. Thermal efficiency

For a solar-driven SAWH, the energy required for desorption can be calculated as

$$Q_{in,H} = \left[\frac{m_w c_{p,s}}{W_d} + m_w c_{p,w} + m_{a,de}\left(c_{p,a} + Y_{s,in} c_{p,v}\right)\right](T_H - T_{cond}) + m_w h_{ad} \tag{S9}$$

where $c_{p,s}$, $c_{p,w}$, $c_{p,a}$ and $c_{p,v}$ are specific heat capacity of sorbent, water, air and vapor, respectively. W_d is water uptake, and T_H and T_{cond} represent the temperature of heating and condensation, and h_{ad} means the desorption enthalpy. The first term of RHS refers to sensible heat consisting of heating energy for sorbent, water and air. The second term of RHS is latent heat.

The parameters of sorbents MOF-303, MOF-801, Al-fumarate, AQSOA Z01 and MOF-841 are summarized in Supporting Table 3. For the calculation of fitting isotherms of S-x (Supporting Figure S8), all thermal properties are uniformed and also included in Supporting Table 3.

The mass of air can be determined by Equation S2, thus air humidity of inlet and outlet in sorbent unit should be provided. Because the inlet of the sorbent unit is the outlet of condensation, the humidity ratio of the sorbent inlet is determined by the condensation temperature, as expressed,

$$Y_{s,de,in} = Y_{cond,out} = Y(T_{cond}, 100\%RH) \tag{S10}$$

The humidity ratio of the sorbent outlet can be derived as

$$Y_{s,de,out} = \frac{\int_{T_{H,min}}^{T_H} Y(RH_{de}, T)\, dT}{\Delta T} \tag{S11}$$

The minimum regeneration temperature $T_{H,min}$ can be calculated as

$$T_{H,min} = T\left(T_{cond}, Y_{cond,out}\right) \tag{S12}$$

Similarly, for CSAWH, besides heating energy for desorption which can be calculated by Equation S9, the cooling capacity of adsorption can be expressed as

$$Q_{in,C} = \left[\frac{m_w c_{p,s}}{W_d} + m_w c_{p,w} + m_{a,ad}\left(c_{p,a} + Y_{s,in} c_{p,v}\right)\right](T_{am} - T_{ad}) + m_w h_{ad} \tag{S13}$$

where T_{ad} is the adsorption temperature, which is assumed as the same as the optimal temperature of the cooling source. The first and second terms of RHS represent the sensible (including heating energy for sorbent, water and air) and latent heat, respectively.

The selection of cooling source should be optimized at the minimum energy consumption as Equation S14. Lower temperature will lead to a higher water uptake but increased energy consumption. Especially when effective RH near the sorbent is higher than step RH, water uptake will not be improved further.

$$T_{ad} = T\{min\ Q_{in,C}\} \tag{S14}$$

And the water uptake is determined by the effective RH and temperature by isotherm, as shown in Equation S15.

$$\begin{cases} W_d = f_{iso}(T_{ad}, RH_{ad}) \\ RH_{ad} = RH(T_{ad}, Y_{ad}) \end{cases} \tag{S15}$$

Assuming the cooling capacity can be provided by a vapor compression cycle with COP of 5[3,4]. Total energy consumption for CSAWH can be expressed as

$$Q_{in} = \frac{Q_{in,C}}{COP \cdot \eta_{PV}} + Q_{in,H} \tag{S16}$$

For a cooling-based AWH, the air needs to be cooled below the dew point. Thus, the outlet air condition is determined as T_{cond} and 100%RH. Then the cooling capacity can be calculated by the enthalpy difference between inlet air and outlet air, as expressed by

$$Q_{c,AWH} = m_a\left(h_{a,in} - h_{a,o}\right) \tag{S17}$$

According to the mass conservation in Equation (S2), the Equation (S17) can be expressed by

$$Q_{c,AWH} = \frac{m_w(h_{a,in} - h_{a,o})}{Y_{a,in} - Y_{a,o}} \tag{S18}$$

Similarly, assuming the vapor compression cycle with COP of 5 is employed[3,4], then the required energy for CAWH can be derived via a PV panel.

S4. Isotherms

For AWH, sorbent with an S-shaped isotherm is an ideal and suitable selection, featuring sorption at high humidity (at night) and desorption at low humidity (at day). Therefore, most AWHs used sorbents with S-shaped isotherms, such as MOF and zeolite. Thus, we discussed water harvesting performance using ideal S-shaped isotherms in this manuscript. The fitting function is shown as follows,

$$W_d = \frac{p/p_0}{1 + \exp(x - 100k)} \tag{S19}$$

where the *x* represents the center position of step pressure, and *k* refers to the equilibrium water uptake. The fitting isotherms can be found in Figure S8.

Considering that the water sorption behavior is widely evaluated by using D-A (Dubibib-Astakhov) equation[5], as shown in Equation S20.

$$W_d = a\exp(b\Delta F) \tag{S20}$$

where *a* and *b* are coefficients, and the *ΔF* is the sorption potential which is the function of temperature and relative pressure, as expressed in Equation S21,

$$\Delta F = RT\, ln\frac{p}{p_0} \tag{S21}$$

where the R is the gas constant. Then we try to convert our fitting isotherms into D-A form as shown in Equation S22.

$$W_d = \frac{1}{1 + \exp(x - 100k)}\exp\left(\frac{\Delta F}{RT}\right) \tag{S22}$$

Here, the coefficient *a* and *b* in D-A equation can be derived as *a*=1/(1+exp(*x*-100*k*)) and *b*=1/R*T*. Therefore, the coefficient *a* is determined by the equilibrium water uptake *k* and step position *x*, and b is determined by the temperature.

S5. Energy balance

Taking a typical passive SAWH as an example, energy conservation for a sorbent unit can be described that receiving incident solar flux (q_{sun}), exchanging heat as conduction, convection and radiation with ambient and condenser, and heating sorbent for desorption (q_{de}). The conductive heat transfer with ambient can be neglected, assuming that area of the solar absorber, sorption and condensation surface are the same, total energy balance can be expressed as

$$q_{sun} = q_{rad,o} + q_{conv,o} + q_{condc,c} + q_{rad,c} + q_{conv,c} + q_{de} \tag{S23}$$

Radiative heat transfer is described by Stefan-Boltzmann law as

$$q_{rad,o} = \varepsilon_s \sigma (T_s^4 - T_\infty^4) \tag{S24}$$

$$q_{rad,c} = \frac{\sigma (T_s^4 - T_c^4)}{\frac{1}{\varepsilon_s} + \frac{1}{\varepsilon_c} - 1} \tag{S25}$$

where ε_s, ε_c and σ are emissivity of sorbent and condenser surface, and Stefan-Boltzmann constant (5.67×10^{-8} W m^{-2} K^{-4}). T_s, T_c and T_∞ are temperature of sorbent, condenser and far-field ambient. Equations S24 and S25 represent the radiative heat transfer for sorbent-ambient and sorbent-condenser surface, respectively.

Convective heat transfer can be calculated by

$$q_{conv,o} = h_{a,o}(T_s - T_\infty) \tag{S26}$$

$$q_{conv,c} = h_{a,i}(T_s - T_c) \tag{S27}$$

where $h_{a,o}$ and $h_{a,i}$ refer to convective heat transfer coefficient (W m^{-2} K^{-1}).

Conductive heat transfer between sorbent and condenser can be regarded by Fourier's law,

$$q_{condc,c} = k\frac{(T_s - T_c)}{b} \tag{S28}$$

where k (W/mK) and b are conductive heat transfer coefficient and distance between sorbent and condenser, respectively.

During the desorption phase, three main processes are included. Absorbing solar energy, the desorption will be trigged under the driven force of humidity difference. Then the water vapor will diffuse to the condenser surface and consequent condensation will be occurred, as described following

$$\dot{m_{de}} = K_y A\left(Y_d - Y_{a,s}\right) \tag{S29}$$

$$\dot{m_{diff}} = D_a\frac{\left(Y_{a,s} - Y_{a,c}\right)}{b} \tag{S30}$$

$$\dot{m_{cond}} = \frac{q_{cond}}{h_{fg}} \tag{S31}$$

In equilibrium, the mass transfer rate of desorption, diffusion and condensation should be the same, thus the heating flux for desorption can be determined as

$$q_{de} = \dot{m_{de}} h_{ad} \tag{S32}$$

Based on the above equations, the energy flow can be described and the heating temperature can be calculated with variable air gap distance.

Besides, heat loss from side wall can be derived as

$$q_{side} = h_{a,side}(T_{side} - T_{\infty}) \tag{S33}$$

The temperature distribution of side wall can estimate liner change. Then the temperature of side wall is expressed as

$$T_{side} = \frac{T_s + T_c}{2} \tag{S34}$$

For an intuitive comparison, heat flux in side wall is transformed the sorbent-area based heat flux as

$$q_{side}^{*} = \frac{4b}{a} q_{side} \tag{S35}$$

Then the net heat flux for condensation can be expressed as

$$q_{c,o} = q_{condc,c} + q_{rad,c} + q_{conv,c} + q_{cond} - q_{side}^{*} \tag{S36}$$

and it should be dissipated by convective heat transfer outsider

$$q_{c,o} = h_{a,c} \frac{A_c}{A} (T_{cond} - T_{\infty}) \tag{S37}$$

Thus, the required area A_c for heat dissipating can be determined by Equation S37.

All parameters could be found in Supporting Table 4.

Supporting Figures

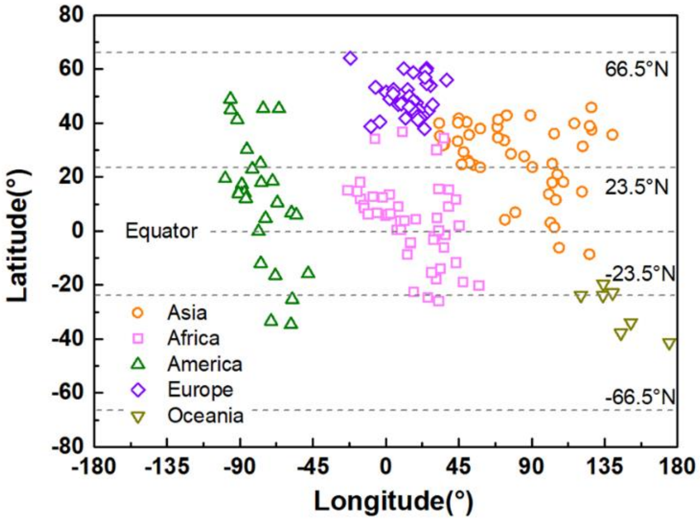

Figure S1. Geographic location of global major cities selected in this work. The weather data is correspondingly mapped in Figure 1b.

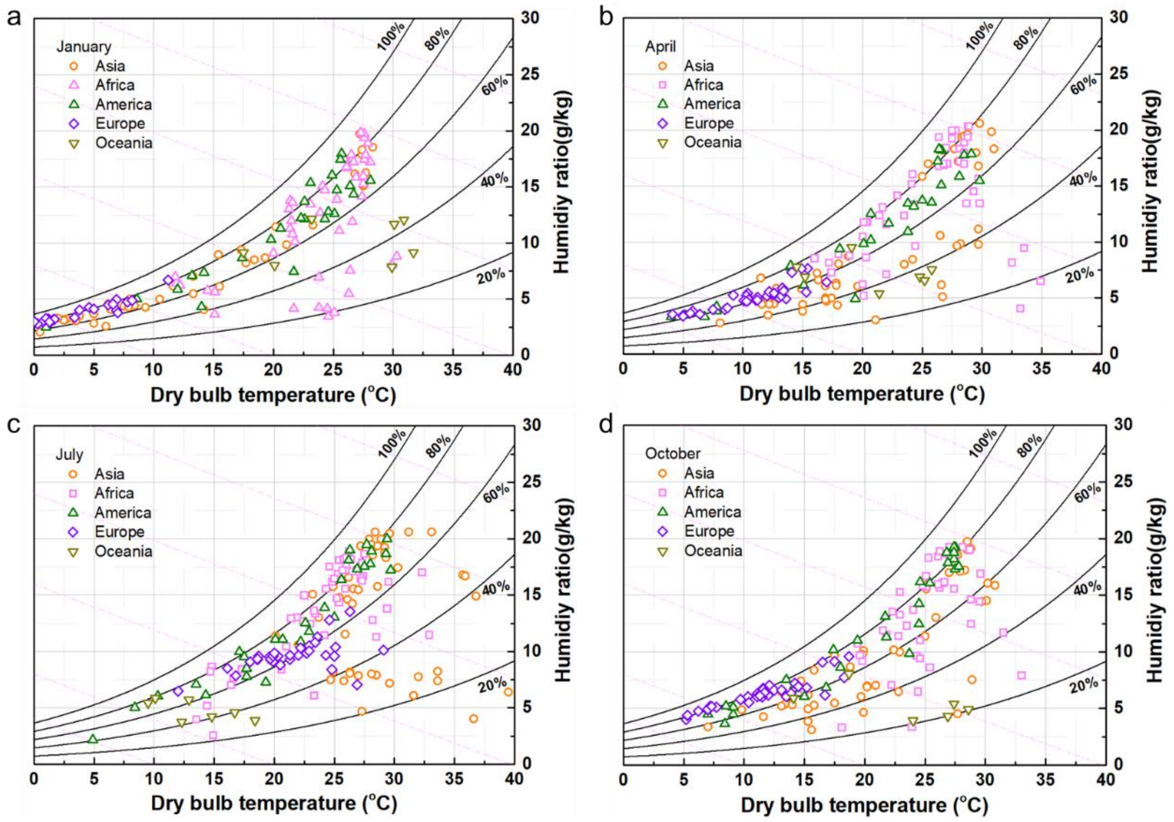

Figure S2. Average weather data of selected global cities on January (a), April (b), July (c) and October (d), which represented the four seasons of the year. The data is from the Meteonorm 8.0.2, and the data is average temperature and humidity based on the contemporary record during 2000-2019.

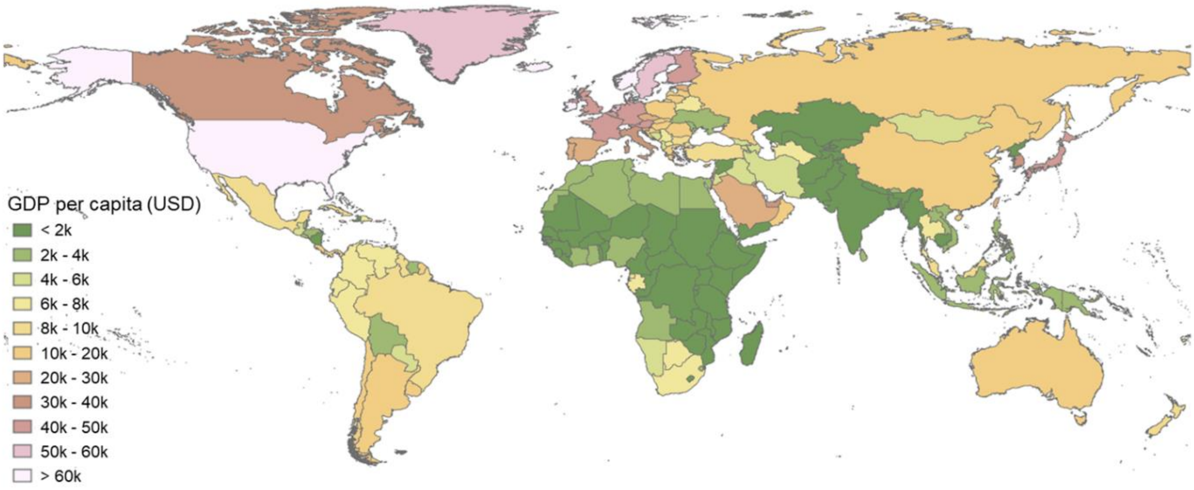

Figure S3. GDP (gross domestic product) per capita (USD) by countries (or regions) in 2019 which are summarized from the website of the ministry of foreign affairs of China.

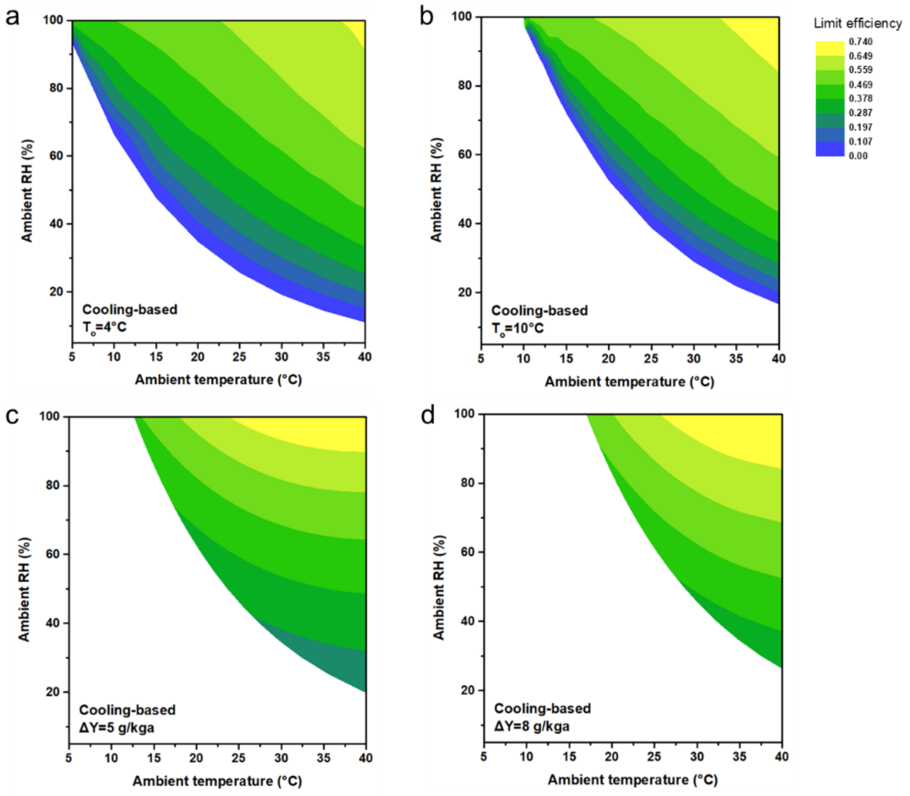

Figure S4. Feasible zone of cooling-based AWH. (a) and (b) are based on the fixed condensation temperature, thus they have a wider feasible zone, but the efficiency will be reduced in the high temperature/humidity zone due to the huge temperature difference between ambient and condensation. (c) and (d) are based on fixed water harvesting rate ($Y_{a,in}$-$Y_{a,o}$, 5g/kg DA and 8g/kg DA), thus the efficiency will be optimal due to the smaller temperature difference.

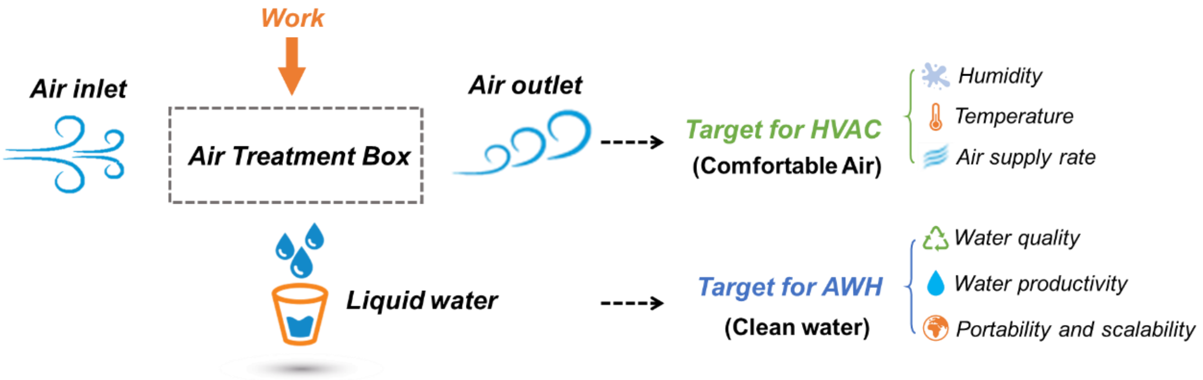

Figure S5. Basic principle of AWH. No matter what technology is used, the air treatment process could be regarded as a box, feeding air, consuming work, and producing air and water. Although the basic work principle of AWH is similar to HVAC (heating, ventilation and air-conditioning), the target is quite different. Comfortable air for HVAC but clean water for AWH.

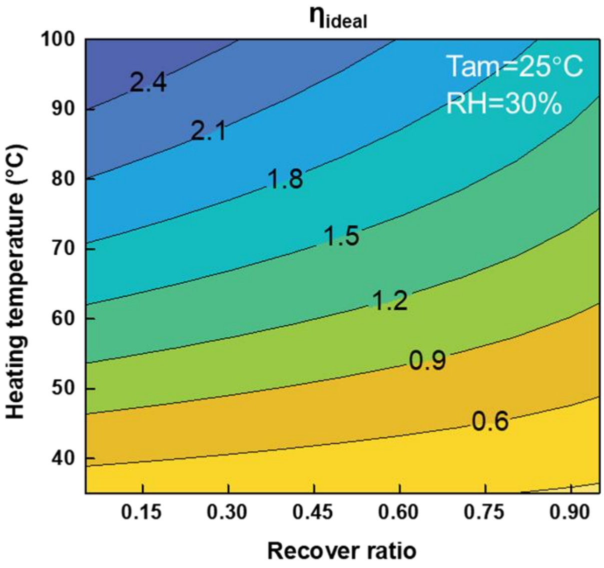

Figure S6. Ideal thermal efficiencies with different heating temperature and racovery ratio.

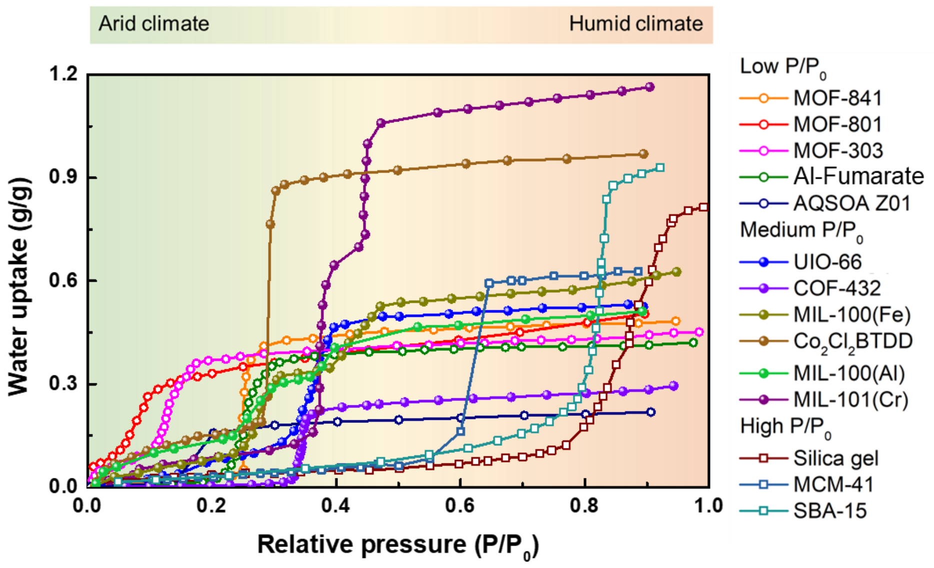

Figure S7. Isotherms of common sorbents. Low P/P_0: MOF-841[6], MOF-801[7], MOF-303[8], Al-fumarate[8] and AQSOA Z01[9]. Medium P/P_0: UiO-66[7], COF-432[10], MIL-100(Fe)[11], Co2Cl2BTDD[12], MIL-100(Al)[13] and MIL-101(Cr)[14]. High P/P_0: Silica gel[15], MCM-41[16] and SBA-15[16].

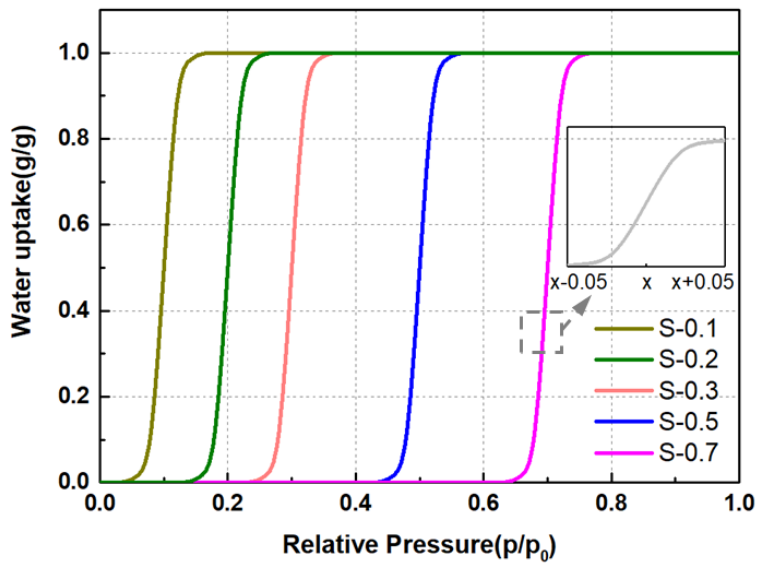

Figure S8. Fitting S-shaped isotherms with different steps, named S-x. (x is the center position of the step range, and the width of the step range is ±0.05)

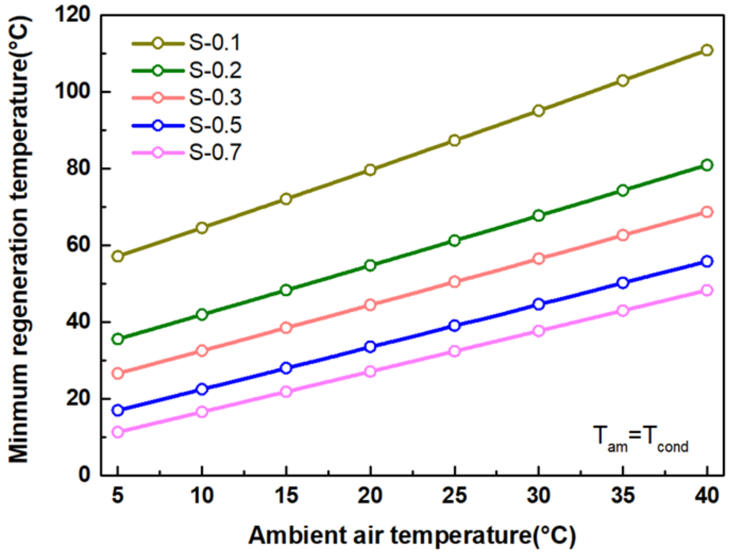

Figure S9. Minimum regeneration temperature for different sorbents. The condensation temperature is assumed as the same as the ambient temperature. Sorbents with later steps have a great potential for low-temperature utilization. The minimum regeneration temperature is conducted considering the same humidity ratio between sorbent and condenser.

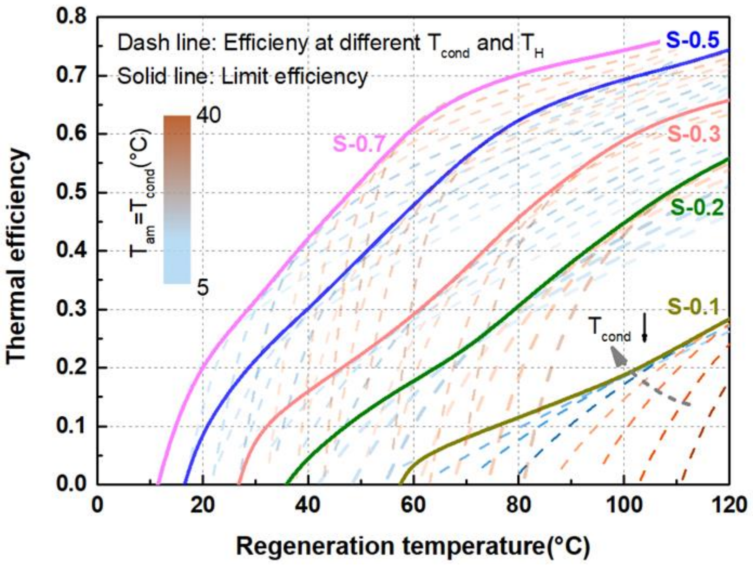

Figure S10. Efficiency limit for different sorbents. Limit efficiency is conducted by fitting the boundary of thermal efficiency under different conditions. The dash line is marked based on the different ambient temperature and humidity should be higher than step RH. Although the sorbents with later steps have higher thermal efficiency, their suitable zone is restricted by the step RH. With the decreasing of condensation temperature, the minimum regeneration temperature is decreasing, but the efficiency will be reduced at higher regeneration temperature due to the larger sensible heat loss.

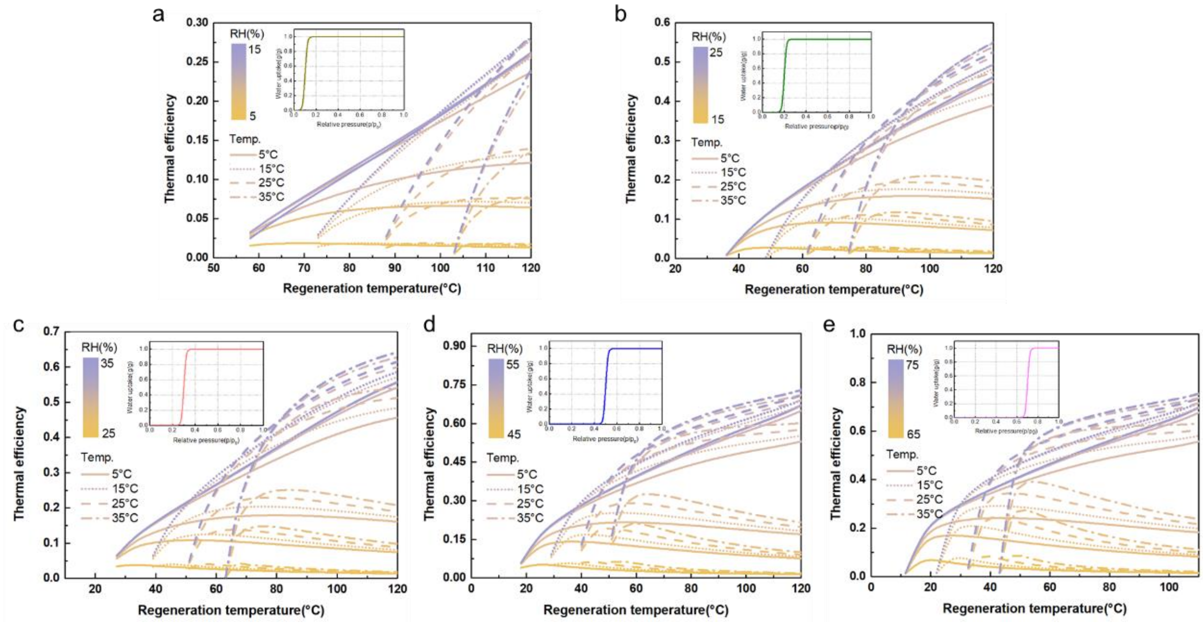

Figure S11. Thermal efficiency for different sorbents with different ambient conditions. (a), (b), (c) and (d) represent the sorbents with step at 0.1, 0.2, 0.3, 0.5 and 0.7, respectively. The lowest RH is limited by the step position (x-0.05). With the RH increasing, thermal efficiency will be firstly increased and then keep constant due to the change of water uptake.

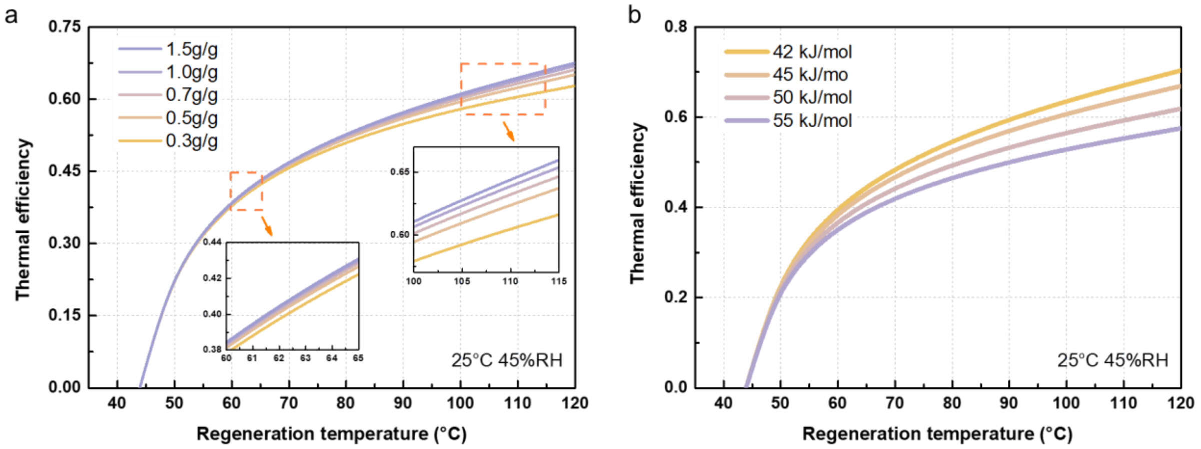

Figure S12. Effect of maximum water uptake and adsorption enthalpy on the thermal efficiency of S-0.4. The ambient condition is 25°C/30%RH. The adsorption enthalpy in Figure S8a is 45 kJ/mol and the maximum water uptake in Figure S8b is 1.0 g/g.

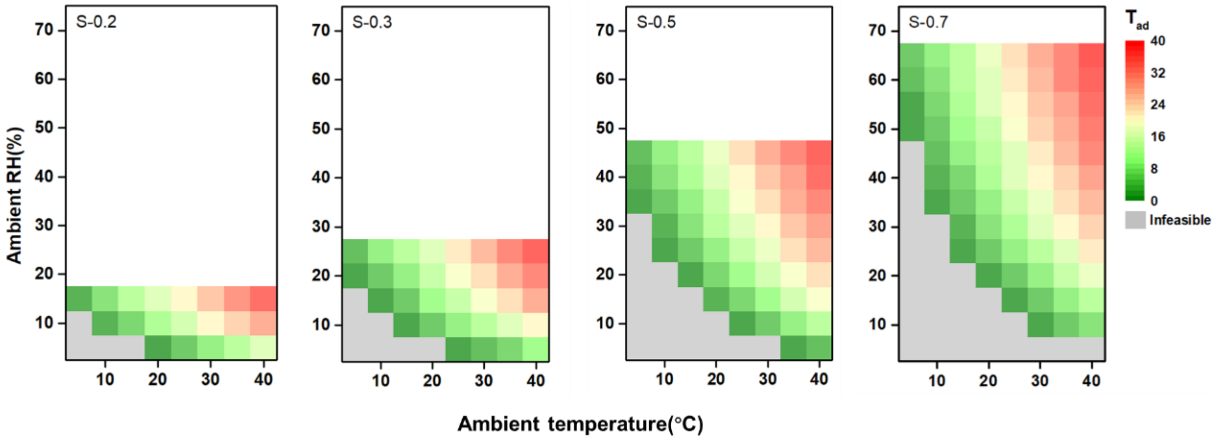

Figure S13. Optimal adsorption temperature for different sorbents via CSAWH. Due to the sorbent S-0.1 can be used in almost all humidity conditions, thus we did not show its performance. The results indicated that higher temperature or humidity will lead to a higher adsorption temperature.

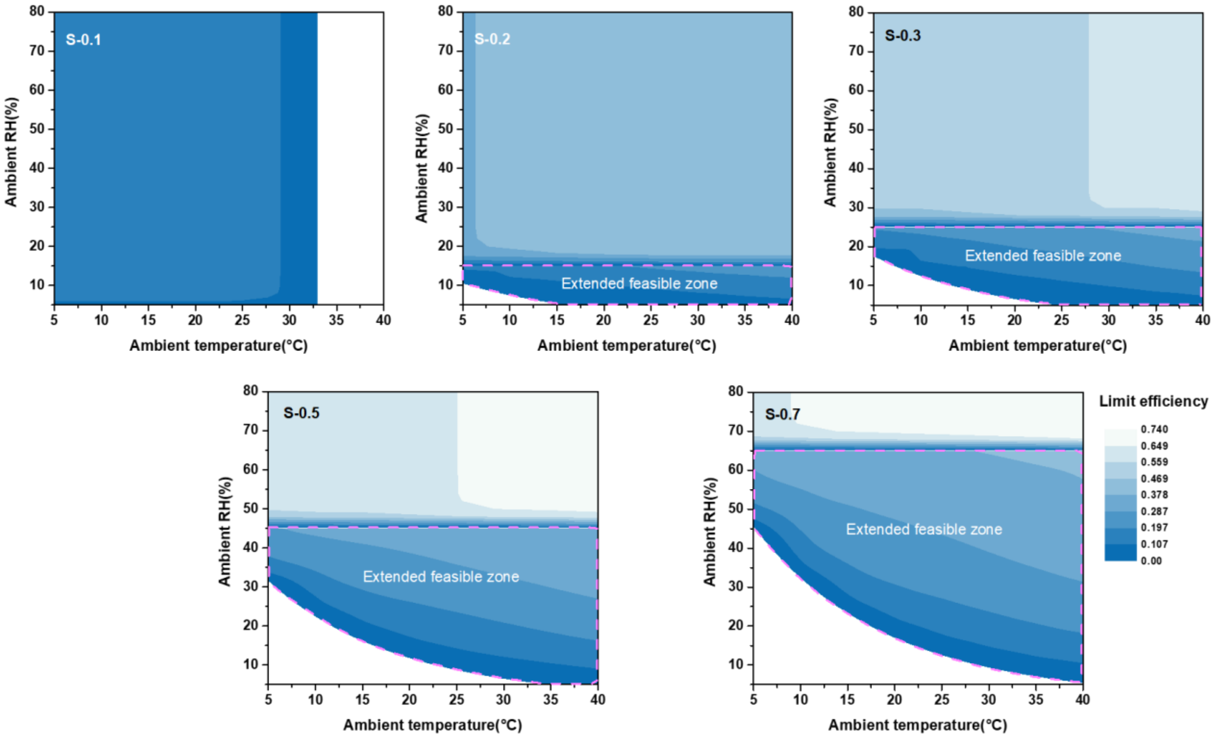

Figure S14. Limit efficiency for CSAWH with different sorbents. The marked zone is extended via using CSAWH, and outside is conventional SAWH. The limit efficiency in the extended zone is determined by the optimal adsorption temperature and a vapor compression cycle is assumed to provide the cooling capacity and then the electricity is converted into solar energy using a PV panel.

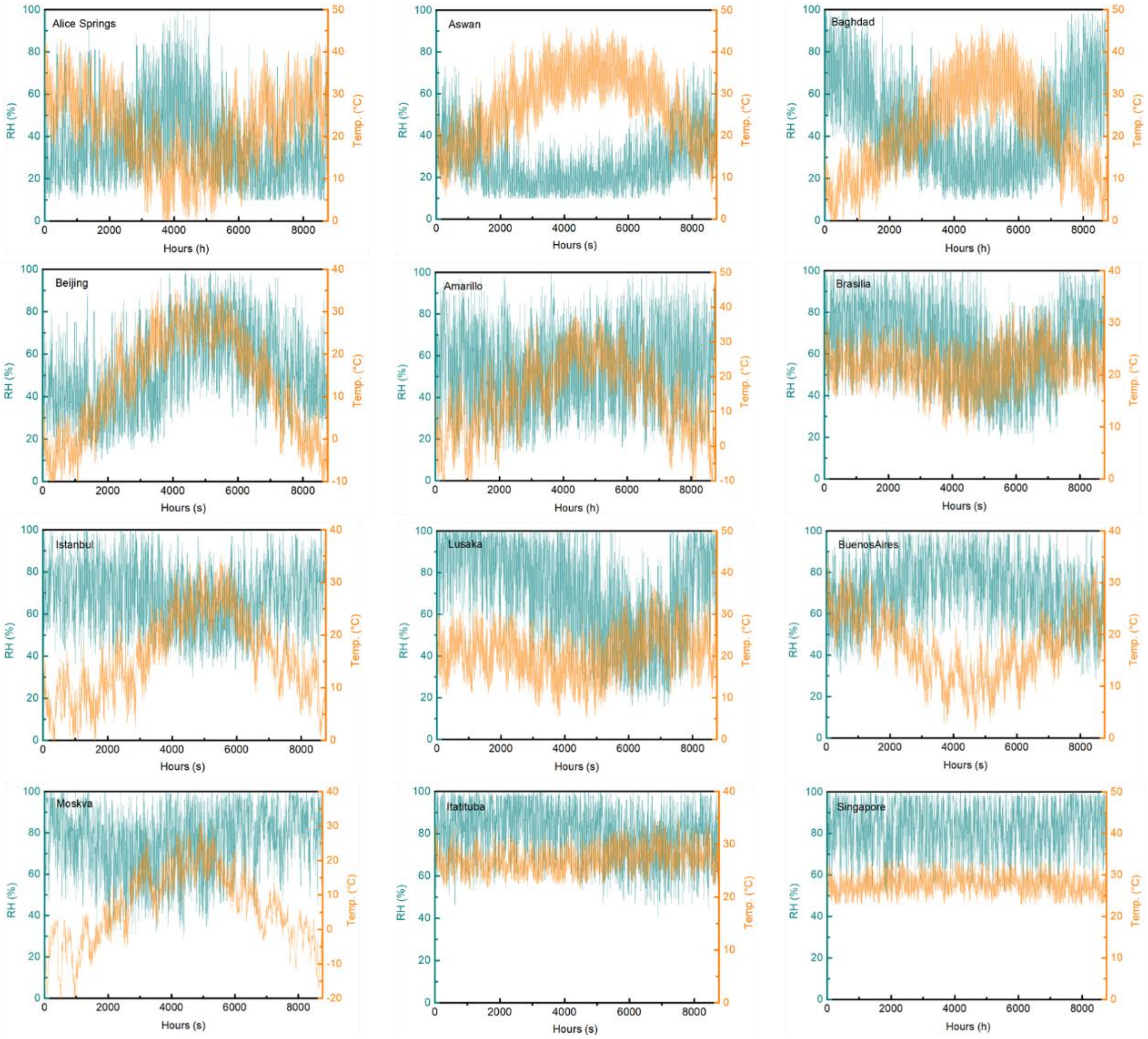

Figure S15. Transient temperature and humidity of several reprehensive cities.

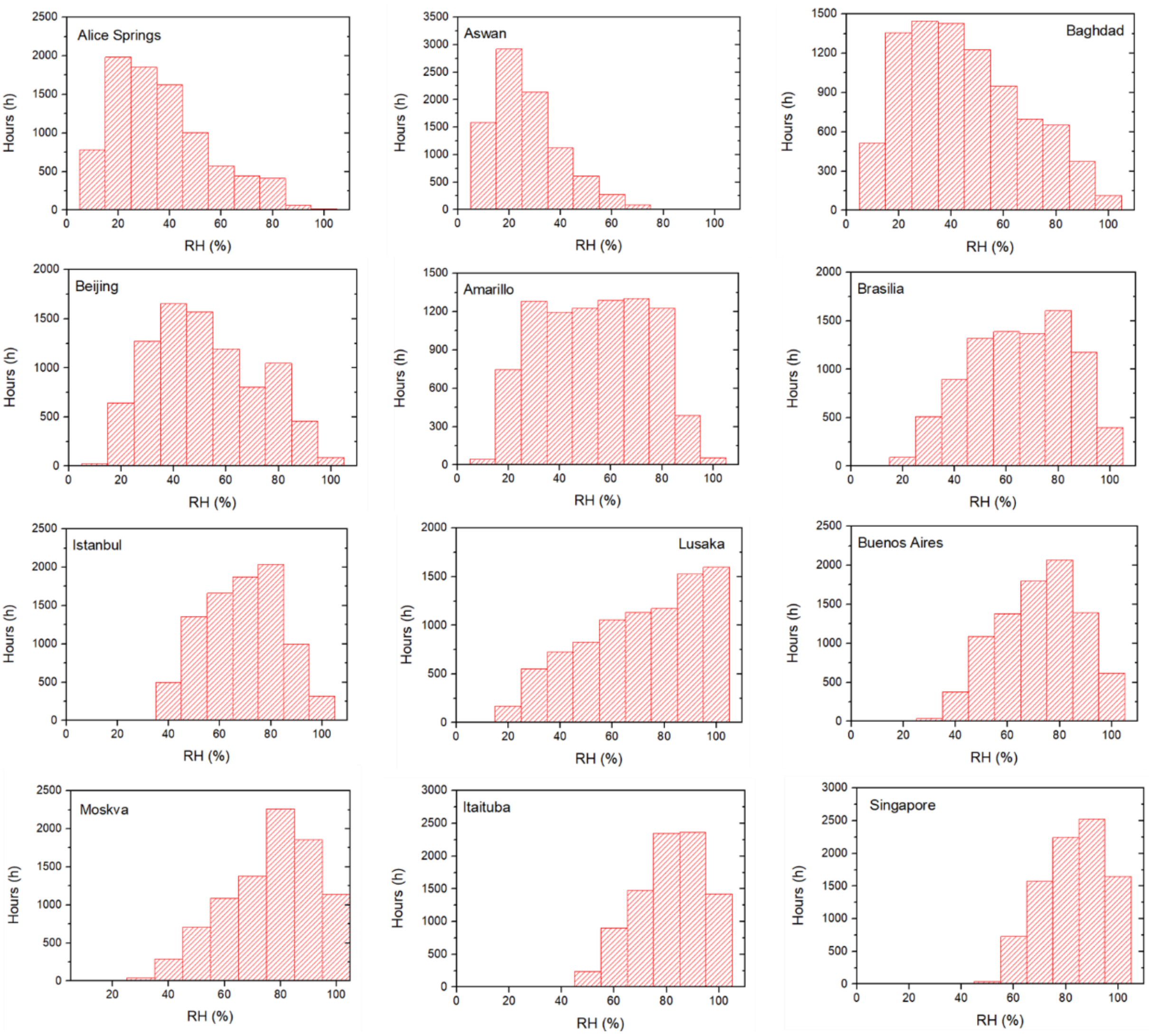

Figure S16. Humidity distribution all around year of several reprehensive cities.

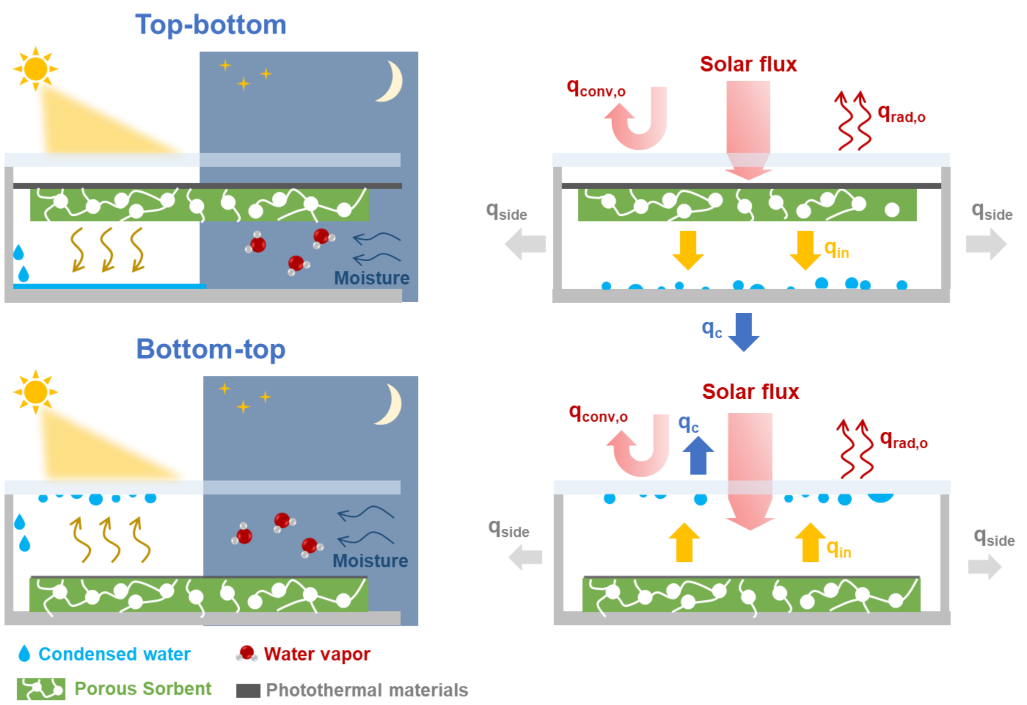

Figure S17. The typical two modes of passive AWH and thier energy flows.

Supporting Tables

Table S1. Summary of release efficiency and condensation efficiency

Ref.	Adsorbed water	Desorbed water	Collected Water	Release efficiency	Condensation efficiency	Water harvesting efficiency
[8]	~1169	~661	562	0.56	0.85	0.48
	~3117	N/A	303	N/A	N/A	0.10
[9]	109	60.5	59.7	0.56	0.98	0.55
[17]	212	83	25	0.39	0.3	0.12
	171	130	56	0.76	0.43	0.33
	90	40	37	0.44	0.93	0.41
	98	85	78	0.86	0.92	0.79
[18]	~75.6	N/A	19.6	N/A	N/A	0.26
[19]	N/A	65.6	46.3	N/A	0.7	N/A
[20]	30	19	16	0.639	0.837	0.535
	19	11.5	8	0.603	0.693	0.418

Note: Release efficiency, condensation efficiency and water harvesting efficiency defined as the ratio of the mass of desorbed water to adsorbed water, the ratio of the mass of collected water to desorbed water and the ratio of the mass of collected water to adsorbed water, respectively.

Table S2. Energy consumption on current AWHs.

Sorbents	Solar Flux (W/m^2)	Solar Area (m^2)	Solar Irradiation Time (h)	Harvested Water (g)	Sorption/ Desorption	Energy consumption (MJ/kg)
LiCl@rGO-SA[19]	1000<br>740	0.02	10	46.3<br>22.8	30°C 57%<br>30°C 56%	15.55<br>23.37
MIL-101@LiCl[21]	1000<br>719	0.0254	1.67<br>1.5	7.0*<br>4.5*	30°C 30%/-	21.77<br>21.92
MOF-801[22]	1000<br>1000	-	3.67<br>1.5	-	25°C 30%/35°C 20%	80(3%)<br>17.14(14%)
MOF-801/G[17]	558<br>792<br>600	0.1552	7.5<br>7.5<br>7.0	37<br>78<br>55	20°C 40%<br>20°C 40%<br>15°C 40% /35°C 5%	63.20<br>42.56<br>42.67
MOF-801[3,7]	1000	-	-	-	25°C 20%	41.67(6%)<br>25(10%)
AQSOA Z01[9]	800	0.078	4.25	60	20°C 68% /-	23.58(10.6%)
HCS-LiCl[18]	600<br>825<br>356	0.0154	1.33<br>1.33<br>2.0	7.7<br>6.5<br>5.4	25°C 65% /-<br>71% /-<br>63% /-	5.75<br>9.37<br>7.3
HCS-LiCl[18]	1000	0.00242	4	1.25	22°C 60% /-	27.88
ILCA[23]	600	0.01766	8	9.116	10°C 70% /25°C 30%	33.48
MOF-303[8]	800[1]	-	-	303.1	27°C 10%	25.87<br>(Heating: 12.54<br>Electricity: 1.59)
ACF/LiCl[24]	-	-	-	14.3×10^3<br>38.5×10^3	25°C 39%<br>25°C 75%	13.89<br>11.83
IMFCA[20]	577	0.005	12	16	22°C 75%/37°C 25%	7.79
ACF/LiCl/PTFE[25]	1000	0.01	4	8.8<br>5.6	22°C 80%	16.36
Bina/FCNT[26]	-	0.005024	-	3.9<br>5.04<br>5.90	20°C 30%	16.2<br>13.3<br>14.1
SHPF[27]	-	-	-	-	15°C 30%	21.09<br>(Heating: 14.76)
ACF/LiCl/CPP[28]	1000	-	6	2.8 L/m^2	25°C 30%	7.714
NBHA[29]	240	0.0144	8	6.66	23°C 70%	14.944
MOF-derived Carbon[30]	1000<br>1000<br>800	0.000962	0.25<br>0.25<br>2.5	0.063<br>0.0288<br>0.4095	30°C 60%<br>23°C 26%<br>28°C 60%	13.737<br>30.05<br>16.91

Table S3. Parameters on thermal efficiency.

Parameter	Value	Parameter	Value
R_a (J/g K)	0.287	R_v (J/g K)	0.4619
$C_{p,ad}$ (J/g K)	1.0	$C_{p,air}$ (J/g K)	1.013
$C_{p,w}$ (J/g K)	4.2	$C_{p,v}$ (J/g K)	2.1
h_{fg} (kJ/mol)	41	h_{ad} (kJ/mol)	45 (default value)
RH_{de} (%)	10 (AQSOA Z01)	h_{ad} (kJ/mol)	45 (AQSOA Z01)
RH_{de} (%)	18 (Al-fumarate)	h_{ad} (kJ/mol)	46 (Al-fumarate)
RH_{de} (%)	18 (MOF-841)	h_{ad} (kJ/mol)	50 (MOF-841)
RH_{de} (%)	7 (MOF-303)	h_{ad} (kJ/mol)	52 (MOF-303)
RH_{de} (%)	5 (MOF-801)	h_{ad} (kJ/mol)	55 (MOF-801)

Table S4. Parameter on energy balance.

Parameter	Value	Parameter	Value
$h_{a,o}$ (W/m^2 K)	10	$h_{a,in}$ (W/m^2 K)	2
$h_{a,side}$ (W/m^2 K)	10	ε_{ab}	0.05
k_a (W/m K)	0.026	ε_{al}	0.05
k_{side} (W/m K)	0.05	ε_{cover}	0.002
t_{side} (m)	0.01	σ (W/m^2 K^4)	5.67×10^{-8}
ρ_{air} (kg/m^3)	1.17	D_g (m^2/s)	3×10^{-5}

Supporting References

[1] M. Alhazmy, *Energy* **2006**, *31*, 2739.

[2] O. Labban, T. Chen, A. F. Ghoniem, J. H. Lienhard, L. K. Norford, *Applied Energy* **2017**, *200*, 330.

[3] H. Kim, S. R. Rao, S. Narayanan, E. A. Kapustin, S. Yang, H. Furukawa, A. S. Umans, O. M. Yaghi, E. N. Wang, *Science* **2017**, *358*, eaao3139.

[4] B. Gido, E. Friedler, D. M. Broday, *Atmospheric Research* **2016**, *182*, 156.

[5] Y. H. Feng, Y. J. Dai, R. Z. Wang, T. S. Ge, *Applied Energy* **2022**, *311*, 118732.

[6] H. Furukawa, F. Gándara, Y.-B. Zhang, J. Jiang, W. L. Queen, M. R. Hudson, O. M. Yaghi, *J. Am. Chem. Soc.* **2014**, *136*, 4369.

[7] H. Kim, S. Yang, S. R. Rao, S. Narayanan, E. A. Kapustin, H. Furukawa, A. S. Umans, O. M. Yaghi, E. N. Wang, *Science* **2017**, *356*, 430.

[8] N. Hanikel, M. S. Prévot, F. Fathieh, E. A. Kapustin, H. Lyu, H. Wang, N. J. Diercks, T. G. Glover, O. M. Yaghi, *ACS Cent. Sci.* **2019**, *5*, 1699.

[9] A. LaPotin, Y. Zhong, L. Zhang, L. Zhao, A. Leroy, H. Kim, S. R. Rao, E. N. Wang, *Joule* **2021**, *5*, 166.

[10] H. L. Nguyen, N. Hanikel, S. J. Lyle, C. Zhu, D. M. Proserpio, O. M. Yaghi, *J. Am. Chem. Soc.* **2020**, *142*, 2218.

[11] S. Cui, M. Qin, A. Marandi, V. Steggles, S. Wang, X. Feng, F. Nouar, C. Serre, *Sci Rep* **2018**, *8*, 15284.

[12] A. J. Rieth, S. Yang, E. N. Wang, M. Dincă, *ACS Cent. Sci.* **2017**, *3*, 668.

[13] F. Jeremias, A. Khutia, S. K. Henninger, C. Janiak, *J. Mater. Chem.* **2012**, *22*, 10148.

[14] Y. Feng, T. Ge, B. Chen, G. Zhan, R. Wang, *Cell Reports Physical Science* **2021**, 100561.

[15] B. Li, L. Hua, Y. Tu, R. Wang, *Joule* **2019**, *3*, 1427.

[16] X. Zheng, T. S. Ge, L. M. Hu, R. Z. Wang, *Ind. Eng. Chem. Res.* **2015**, *54*, 2966.

[17] F. Fathieh, M. J. Kalmutzki, E. A. Kapustin, P. J. Waller, J. Yang, O. M. Yaghi, *Sci. Adv.* **2018**, *4*, eaat3198.

[18] R. Li, Y. Shi, M. Wu, S. Hong, P. Wang, *Nano Energy* **2020**, *67*, 104255.

[19] J. Xu, T. Li, T. Yan, S. Wu, M. Wu, J. Chao, X. Huo, P. Wang, R. Wang, *Energy Environ. Sci.* **2021**, *14*, 5979.

[20] F. Deng, C. Xiang, C. Wang, R. Wang, *J. Mater. Chem. A* **2022**, *10*, 6576.

[21] J. Xu, T. Li, J. Chao, S. Wu, T. Yan, W. Li, B. Cao, R. Wang, *Angew. Chem. Int. Ed.* **2020**, *59*, 5202.

[22] H. Kim, S. R. Rao, E. A. Kapustin, L. Zhao, S. Yang, O. M. Yaghi, E. N. Wang, *Nat*

Commun **2018**, *9*, 1191.

[23] F. Deng, C. Wang, C. Xiang, R. Wang, *Nano Energy* **2021**, *90*, 106642.

[24] J. Y. Wang, R. Z. Wang, Y. D. Tu, L. W. Wang, *Energy* **2018**, *165*, 387.

[25] H. Shan, Q. Pan, C. Xiang, P. Poredoš, Q. Ma, Z. Ye, G. Hou, R. Wang, *Cell Reports Physical Science* **2021**, *2*, 100664.

[26] A. Entezari, M. Ejeian, R. Wang, *ACS Materials Lett.* **2020**, *2*, 471.

[27] Y. Guo, W. Guan, C. Lei, H. Lu, W. Shi, G. Yu, *Nat Commun* **2022**, *13*, 2761.

[28] Y. Wang, S. Gao, H. Zhong, B. Zhang, M. Cui, M. Jiang, S. Wang, Z. Wang, *Cell Reports Physical Science* **2022**, 100879.

[29] M. Wang, T. Sun, D. Wan, M. Dai, S. Ling, J. Wang, Y. Liu, Y. Fang, S. Xu, J. Yeo, H. Yu, S. Liu, Q. Wang, J. Li, Y. Yang, Z. Fan, W. Chen, *Nano Energy* **2021**, *80*, 105569.

[30] Y. Song, N. Xu, G. Liu, H. Qi, W. Zhao, B. Zhu, L. Zhou, J. Zhu, *Nat. Nanotechnol.* **2022**, DOI 10.1038/s41565-022-01135-y.
